# *Drosophila* Ror is a nervous system-specific co-receptor for Wnt ligands

**DOI:** 10.1242/bio.033001

**Published:** 2018-10-19

**Authors:** Caroline Ripp, Julia Loth, Iveta Petrova, Karen Linnemannstöns, Monique Ulepic, Lee Fradkin, Jasprien Noordermeer, Andreas Wodarz

**Affiliations:** 1 Stem Cell Biology, Institute for Anatomy and Cell Biology, Georg-August University Göttingen, Justus-von-Liebig-Weg 11, 37077 Göttingen, Germany; 2 Laboratory of Developmental Neurobiology, Department of Molecular Cell Biology, Leiden University Medical Center, Einthovenweg 20, 2300RC Leiden, The Netherlands; 3 Molecular Cell Biology, Institute I for Anatomy, University of Cologne Medical School, Kerpener Str. 62, 50937 Köln, Germany; 4 Department of Neurobiology, University of Massachusetts Medical School, 364 Plantation Street, LRB 760, Worcester, MA 01605, USA; 5 Cluster of Excellence - Cellular stress response in aging-associated diseases (CECAD), University of Cologne, Joseph-Stelzmann-Str. 26, 50931 Cologne, Germany

**Keywords:** Ror, Wnt signaling, Nervous system development, Planar cell polarity

## Abstract

Wnt ligands are secreted glycoproteins that control many developmental processes and are crucial for homeostasis of numerous tissues in the adult organism. Signal transduction of Wnts involves the binding of Wnts to receptor complexes at the surface of target cells. These receptor complexes are commonly formed between a member of the Frizzled family of seven-pass transmembrane proteins and a co-receptor, which is usually a single-pass transmembrane protein. Among these co-receptors are several with structural homology to receptor tyrosine kinases, including Ror, PTK7, Ryk and MUSK. In vertebrates, Ror-2 and PTK7 are important regulators of planar cell polarity (PCP). By contrast, PCP phenotypes were not reported for mutations in *off-track* (*otk*) and *off-track2* (*otk2*), encoding the *Drosophila* orthologs of PTK7. Here we show that *Drosophila* Ror is expressed in the nervous system and localizes to the plasma membrane of perikarya and neurites. A null allele of *Ror* is homozygous viable and fertile, does not display PCP phenotypes and interacts genetically with mutations in *otk* and *otk2*. We show that Ror binds specifically to Wingless (Wg), Wnt4 and Wnt5 and also to Frizzled2 (Fz2) and Otk. Our findings establish *Drosophila* Ror as a Wnt co-receptor expressed in the nervous system.

## Abstract (precis)

**Summary:** *Drosophila* Ror is a Wnt co-receptor expressed in the nervous system. A *Ror* null mutant allele is viable and shows genetic interaction with mutations in *off-track* and *off-track2*.

## INTRODUCTION

Secreted proteins of the Wnt family are among the most potent regulators of development and tissue homeostasis (Angers and Moon, 2009; van Amerongen and Nusse, 2009; Wodarz and Nusse, 1998). In humans, 19 Wnt genes have been identified that differ greatly in their expression pattern and function (van Amerongen and Nusse, 2009). Wnt proteins trigger intracellular signal transduction cascades by binding to receptor complexes at the cell surface. These receptor complexes usually consist of a member of the Frizzled (Fz) family of seven-pass transmembrane proteins and a co-receptor, commonly a single-pass transmembrane protein (Angers and Moon, 2009; Niehrs, 2012). There is ample evidence that Wnts, Fz receptors and Wnt co-receptors are quite promiscuous in their binding specificities and that the signaling outcome is mostly dictated by the specific combination of Wnt ligand, Fz receptor and Wnt co-receptor coming together on the surface of a specific cell type. Wnt signaling pathways have been ordered into several categories, including the beta-catenin-dependent Wnt pathway (also sometimes called the canonical Wnt pathway), the Wnt-planar cell polarity (PCP) pathway and the Wnt-Ca^2+^ pathway (Angers and Moon, 2009; van Amerongen and Nusse, 2009). One of the biggest questions in the field is how a certain Wnt protein can trigger only one of these downstream pathways while another Wnt may trigger a different pathway in the same cell type.

It has recently been shown that Wnt co-receptors of the Ror family of single-pass transmembrane tyrosine kinases promote signaling via the Wnt-PCP pathway and suppress beta-catenin-dependent Wnt signaling (Green et al., 2014; Mikels and Nusse, 2006; Oishi et al., 2003). Mice, frogs and zebrafish mutant for Ror2 display strong PCP phenotypes characterized by defects in convergent-extension movements, directed cell migration and the orientation of hair cells in the inner ear in mice (Bai et al., 2014; DeChiara et al., 2000; Minami et al., 2009; Petrova et al., 2013; Schambony and Wedlich, 2007; Takeuchi et al., 2000; Yamamoto et al., 2008). These phenotypes are very similar to those reported for another Wnt co-receptor, protein tyrosine kinase 7 (PTK7) (Hayes et al., 2013; Lu et al., 2004; Shnitsar and Borchers, 2008). Indeed, both proteins were shown to bind to each other and to cooperate in Wnt-PCP signaling (Martinez et al., 2015; Podleschny et al., 2015).

Surprisingly, mutations in the *Drosophila* homologs of PTK7 called Off-track (Otk) and Off-track2 (Otk2) do not display PCP phenotypes in wings, eyes or in the adult epidermis, but instead lead to male sterility caused by morphogenesis defects of the ejaculatory duct (Linnemannstöns et al., 2014). For the two *Drosophila* Ror homologs Ror and Neurospecific receptor kinase (Nrk), no functional data have been published so far, nor is the expression pattern and subcellular localization of the two Ror-related proteins known.

Here we present the detailed expression pattern and subcellular localization of a Ror-eGFP fusion protein expressed under control of the endogenous *Ror* promoter region. The corresponding fosmid construct was generated by recombineering in bacteria followed by stable chromosomal integration into the genome of transgenic flies (Venken et al., 2008). The expression analysis revealed that Ror is expressed in neuroblasts and in the majority, if not all, of CNS and PNS neurons, but not in glia cells. The protein is localized to the plasma membrane of cell bodies and axons of neurons and is detectable in the postsynaptic membrane of larval neuromuscular junctions (NMJs). We have generated a deletion allele of *Ror* that lacks the translation start site, the signal peptide and large parts of the region encoding the extracellular domain and thus is predicted to be a functional null allele. This allele is homozygous viable and does not cause any major defects in CNS development. As reported for *otk* and *otk2*, loss of *Ror* function does not cause PCP defects. However, the *Ror* null allele interacts genetically with mutations in *otk* and *otk2*, strongly indicating that *Drosophila* Ror is a component of Wnt signal transduction. This hypothesis is corroborated by our finding that Ror binds specifically to the Wnt ligands Wingless (Wg), Wnt4 and Wnt5, as well as to the Wnt receptors Fz2 and Otk.

Together, our data reveal that *Drosophila* Ror is a bona fide Wnt co-receptor expressed predominantly in the nervous system that may function together with Otk and Otk2.

## RESULTS

### Expression pattern of Ror-eGFP

The expression pattern of *Drosophila Ror* has previously been described at the transcript level. *Ror* transcripts have been observed in the embryonic brain, the CNS and in additional cells in the head and trunk of embryos (Wilson et al., 1993). To investigate the expression pattern at the protein level and its subcellular localization, we generated a fly line expressing a Ror-eGFP fusion protein under control of the endogenous *Ror* promoter (Ror-eGFP).

### Ror-eGFP is expressed in the embryonic nervous system

**Fig. 1.**
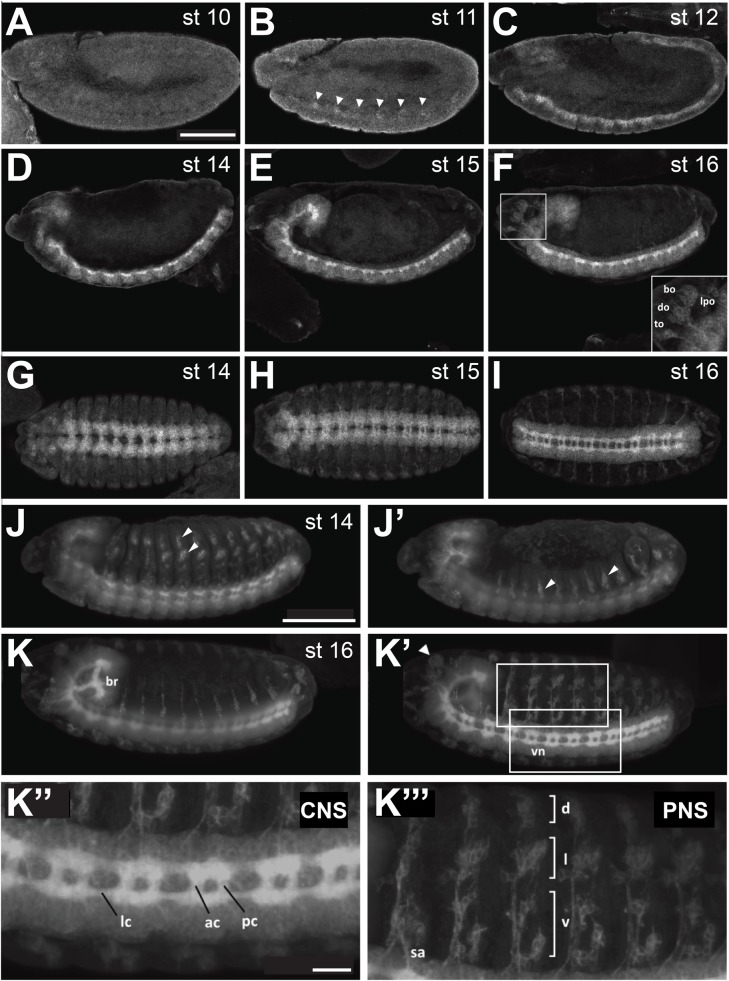
**Expression of a Ror-eGFP fusion protein under control of the endogenous *Ror* promoter in *Drosophila* embryos.** (A-F) Lateral views of stage 10-12 and 14-16 embryos. (G-I) Stage 14-16 embryos viewed from the ventral side, anterior to the left. (J-K‴) Light sheet fluorescence microscopy images of Ror-eGFP embryos. The images show maximum intensity projections of stacks taken from whole embryos. (J,J′) and (K,K′) show the same embryo, respectively, scanned from both sides. (J,J′) At stage 14 Ror-eGFP expression is strong in the embryonic CNS and already visible in the developing PNS. (K,K′) At stage 16 Ror-eGFP is expressed throughout the entire nervous system. (K″) Enlarged view of the CNS seen in (K′). (K‴) Enlarged view of the PNS seen in (K′). Areas shown at higher magnification in (K″) and (K‴) are indicated by boxes in (K′). Scale bars for A-K′: 100 µm; K″,K‴: 20 µm. bo, bolwig's organ; do, dorsal organ; to, terminal organ; lpo, lateropharyngeal organ; br, embryonic brain; vn, ventral nerve cord; lc, longitudinal connectives; ac, anterior commissures; pc, posterior commissures; sa, sensory axon; d, dorsal cluster; l, lateral cluster; v, ventral cluster. In (A-F) and (J-K′) anterior is to the left and dorsal up. Arrowheads in (B) point to the first cell clusters showing Ror-eGFP expression during embryonic development. Arrowheads in (J, J′ and K′) point to sensilla of the PNS.

To analyze the expression pattern and subcellular localization of Ror, we stained embryos expressing the Ror-eGFP fusion protein with an anti-GFP antibody. The protein was first detected at developmental stage 11 when the germ band is fully elongated (Fig. 1B, arrowheads). At this stage Ror-eGFP was visible in segmentally repeated groups of cells. The expression level was initially weak but increased in successive stages and persisted throughout embryonic development (Fig. 1B-F). After completion of germ band retraction, the protein was strongly expressed in the embryonic ventral nerve cord and in the brain (Fig. 1D) and became more prominent as the ventral nerve cord condensed into its final ladder-like structure (Fig. 1E-I). Ror-eGFP was not only expressed in the plasma membrane of neuronal cell bodies (perikarya), but also in their axonal processes forming the commissures and connectives of the ventral nerve cord (Fig. 1I,K′,K″). While it was shown that expression of Otk and Otk2 were both enriched on axons forming the anterior commissures when compared to the posterior commissures (Linnemannstöns et al., 2014), this was not the case for Ror-eGFP. The intensity of the GFP signal was evenly distributed throughout the ventral nerve cord (Fig. 1K″).

In addition to the expression in the central nervous system, Ror-eGFP was also expressed in the sensory cells of the embryonic peripheral nervous system (PNS) from developmental stage 13 onwards (Fig. 1J-K′,K‴). At stage 14 the sensilla which appear in the first wave of differentiation expressed Ror-eGFP (Fig. 1J,J′, arrowheads). In the abdominal segments of stage 16 embryos, Ror-eGFP was observed at the cell membrane of neurons in all three clusters of sensory organs of the PNS (Fig. 1K′,K‴) including the sensory axons which connect to the CNS. In addition, Ror-eGFP was expressed in the larval head sensory organs (Fig. 1F, inset; K′, arrowhead): the Bolwig's organ, which represents the larval eye, and also in the dorsal, terminal and lateropharyngeal organs, all performing gustatory functions.

To determine whether Ror-eGFP is expressed in all cells of the central nervous system or only in a certain subset, we stained embryos with the neuroblast marker Miranda (Mira) and the neuronal marker Elav. In the ventral nerve cord of a stage 16 embryo Ror-eGFP localized to the membrane of at least the vast majority and maybe all neurons, marked with Elav (Fig. S1A). At higher magnification, expression was also detected at the membrane of dividing neuroblasts (Fig. S1A′, asterisk).

### Ror-eGFP is expressed throughout the larval nervous system

**Fig. 2.**
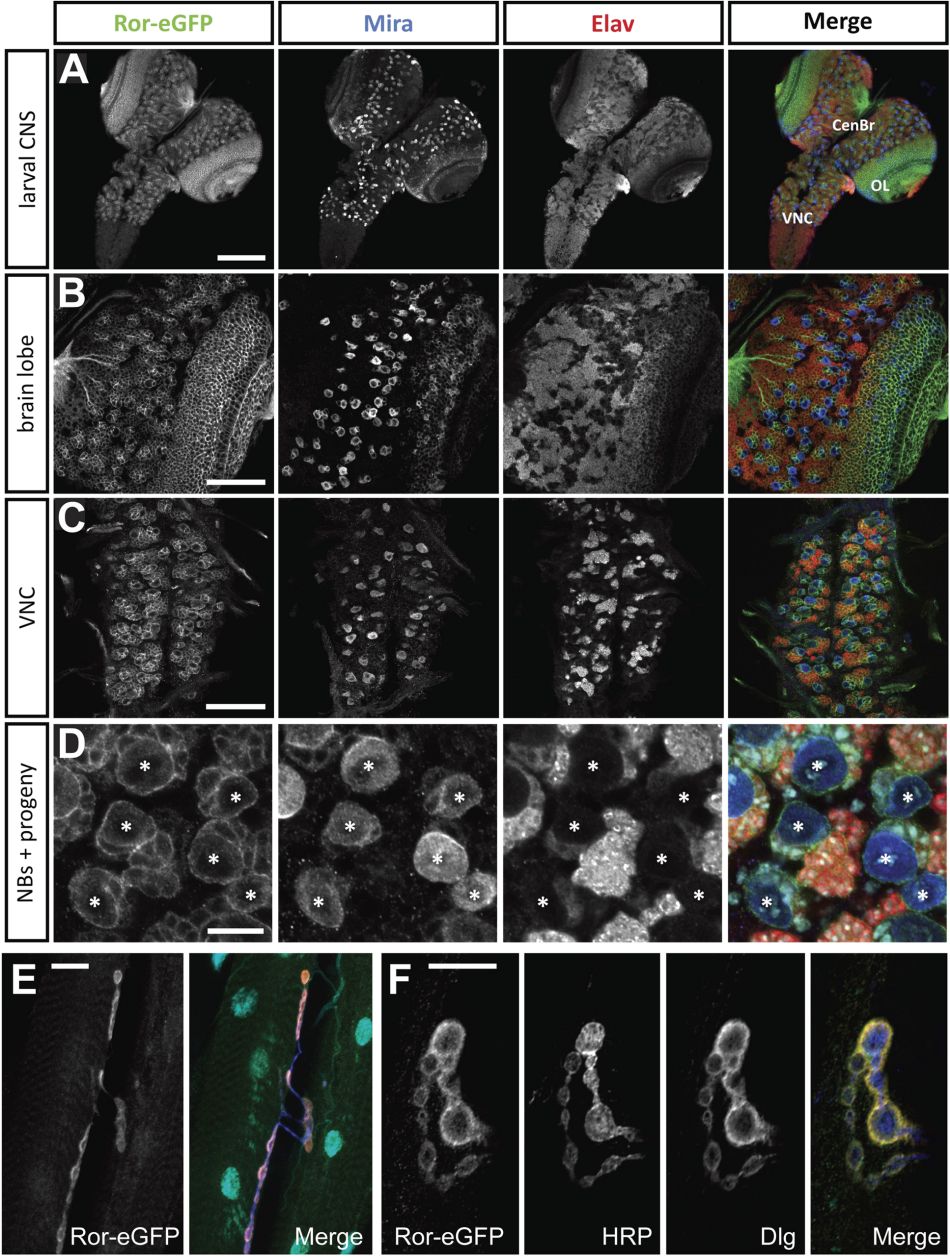
**Ror-eGFP expression in the central nervous system and body wall muscles of third instar larvae.** (A) Overview of the larval central nervous system. Ror-eGFP is expressed in neuroblasts (NBs) and their neuronal progeny. (B) Larval brain lobe. (C) Larval ventral nerve cord. (D) Magnification of a section of a larval brain lobe including NBs, neurons and most likely also GMCs. In (D) the merged image additionally contains Hoechst staining (cyan) labeling the DNA. (E) Neuromuscular junctions (NMJs) of third instar larval body wall muscles. (F) High magnification image of NMJ boutons. The merged images in (E) and (F) show Ror-eGFP in green, HRP in blue, Dlg in red and DAPI in cyan. Scale bars: A, 100 µm; B,C, 50 µm; E, 20 µm; D,F, 10 µm. CenBr, central brain; OL, optic lobe; VNC, ventral nerve cord; *, NB.

In order to analyze the expression pattern of Ror-eGFP in the larval CNS, we dissected larval brains from third instar larvae and stained them for GFP, Mira and Elav. Ror-eGFP expression was detected throughout the larval CNS including the central brain, the optic lobe and the ventral nerve cord (Fig. 2A). The expression in the larval brain was much stronger compared to the expression in embryos. In all parts of the larval brain, expression was detected in the plasma membrane of neuroblasts and in their neuronal progeny marked by Elav (Fig. 2B-D).

To investigate whether Ror-eGFP expression is restricted to neuronal cells, we stained larval brains for the glial marker Reversed polarity (Repo; Fig. S2). While Ror-eGFP was detectable in the membrane of most, and maybe all neuronal cells, we could not detect expression in Repo-positive glia cells (Fig. S2B, asterisks).

### Ror-eGFP is expressed in the neuromuscular junction of larval body wall muscles and in larval imaginal discs

To investigate whether Ror is exclusively expressed in the nervous system or is also expressed in muscles, we dissected larval body wall muscles and stained for GFP (to detect Ror-eGFP), the presynaptic marker anti-HRP and the postsynaptic marker Dlg. We detected strong expression of Ror-eGFP in the NMJs of these muscles (Fig. 2E). High magnification Airyscan imaging of synaptic boutons revealed that Ror-eGFP colocalized with Dlg and showed little overlap with anti-HRP immunofluorescence, indicating that Ror is expressed in the postsynaptic membrane of somatic muscles (Fig. 2F).

Ror-eGFP expression was also detected in third instar larval imaginal discs. In wing discs, haltere discs, leg discs, eye-antennal discs and genital discs Ror-eGFP was expressed in small subsets of cells. In wing imaginal discs the protein was visible in one cell cluster in a region of the disc corresponding to the adult ventral wing surface and in a row of smaller cell clusters along the proximal-distal axis of the disc (Fig. S3A). These cells likely represent proneural clusters or specified sensory organ precursor cells (SOPs). In haltere imaginal discs, Ror-eGFP was expressed in several cells in the proximal part of the disc (Fig. S3B). In eye-antennal discs, Ror-eGFP was found in the developing photoreceptor cells and in the antennal portion of the eye-antennal disc. There, it was located in a cell cluster that may correspond to sensory cells of the Johnston's organ, an auditory organ in the antenna (Fig. S3C). In leg imaginal discs, Ror-eGFP was expressed in one bigger cell cluster probably representing sensory cells of the femoral chordotonal organ and in several smaller cell clusters within the disc (Fig. S3D). Ror-eGFP expression in genital discs, which later form the terminalia (genitalia and analia), was observed in four distinct cell clusters in the female genital disc (Fig. S3E).

### Expression of Ror-eGFP is not affected by mutations in *Wnt* genes

For Otk it was shown that its expression was strongly reduced in embryos homozygous mutant for *Drosophila Wnt2* (Fig. S4C) (Linnemannstöns et al., 2014). This finding pointed to Otk being a post-transcriptional target of Wnt2 signaling. Interestingly, a corresponding effect was not observed for expression of Otk2 (Linnemannstöns et al., 2014). In order to analyze whether the expression of Ror was also regulated by Wnt signaling, we crossed the Ror-eGFP transgene into several *Drosophila Wnt* mutant lines and compared the GFP expression in heterozygous (Fig. S4A) and homozygous mutant embryos (Fig. S4B-E). Neither mutation of *wg* (Fig. S4B), nor of *Wnt2* (Fig. S4C), *Wnt4* (Fig. S4D) or *Wnt5* (Fig. S4E) had any effect on the expression of Ror-eGFP, whereas the reduction of Otk in the CNS was confirmed in homozygous *Wnt2* mutant embryos (Fig. S4C). Note that although the morphology of homozygous *wg*^CX4^ mutant embryos was so severely disturbed that the developmental stage could not be precisely determined, the Ror-eGFP signal in the CNS was clearly detectable and was comparable in intensity to the other genotypes analyzed (Fig. S4B).

### Generation of a null allele for *Ror*

**Fig. 3.**
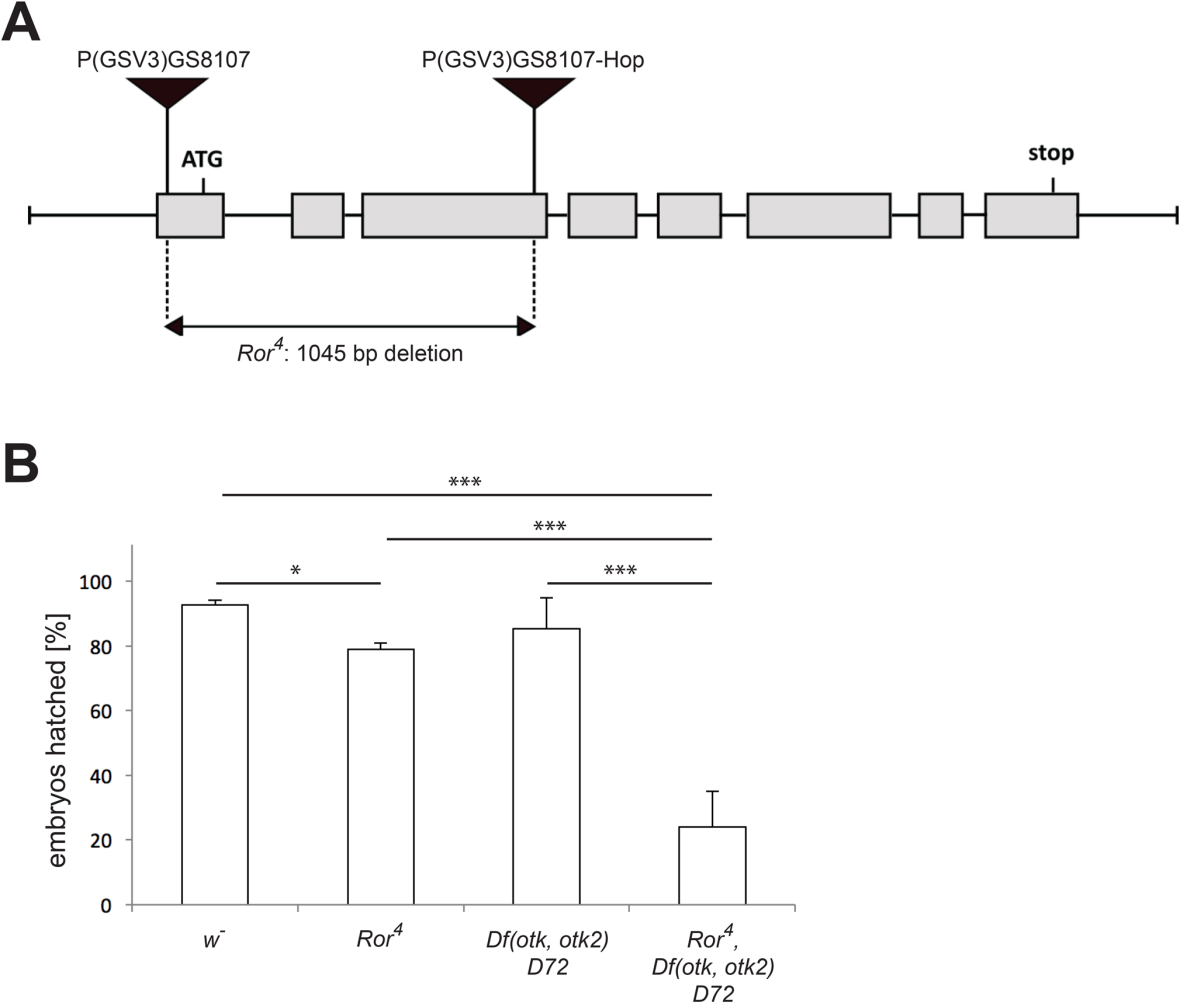
**Generation of the null allele *Ror^4^*.** (A) The exon-intron structure of the *Ror* locus including the positions of the ATG and stop codons is shown. The P-element P{GSV3}GS8107 was first mobilized using P-transposase and re-integrated into the third exon of the Ror coding region (P{GSV3}GS8107-Hop). Then the region between the two P-elements was excised in a second transposase-mediated step. (B) Embryonic viability assay. Data were obtained by repeating each experiment at least three times. The error bars represent the standard deviation of the mean. *P<0.05; ***P<0.001 (independent samples Student's *t*-test).

In vertebrates and *C. elegans*, Ror proteins are involved in many processes during development, including skeletal and neuronal development. To investigate the function of *Drosophila* Ror during development, we generated a null allele for *Ror*. The *Ror^4^* deletion was generated by first mobilizing the P-element P{GSV3}GS8107 located in the *Ror* 5′ UTR, followed by isolation of a line with the reintegration of the P-element in the coding region of *Ror* (P{GSV3}GS8107-Hop). Subsequently, the genomic region in between the two P-elements was removed by transposase-mediated excision. With this approach, 1045 bp of genomic DNA have been removed (chromosome 2L positions 10251861-10252906), including the *Ror* start codon and most of the first three exons (Fig. 3A). Due to the lack of the start codon and the signal peptide, the generated fly line likely represents a null allele. *Ror^4^* mutant flies are homozygous viable and fertile.

### *Ror^4^* shows genetic interaction with mutations in *otk* and *otk2*

Because of the similarities in the expression patterns of Ror, Otk and Otk2 in the nervous system (Linnemannstöns et al., 2014) and their related protein structure, we speculated that the three genes may function in a redundant manner. To address this, we generated by meiotic recombination the triple mutant chromosome *Ror^4^*, *Df(otk,otk2)D72* carrying null alleles of all three genes. Embryonic hatching rates for *Ror^4^*, *Df(otk,otk2)D72* double mutant, *Ror^4^*, *Df(otk,otk2)D72* triple mutant and a *w^−^* control were determined (Fig. 3B). When compared to the *w^−^* control, homozygous *Ror^4^* mutant embryos displayed a slight but significant increase in embryonic lethality. The *Df(otk,otk2)D72* double mutant did not show a significant reduction of embryonic viability compared to the *w^−^* control. By contrast, the embryonic viability of homozygous *Ror^4^*, *Df(otk,otk2)D72* triple mutants was strongly reduced when compared to all three other genotypes (Fig. 3B), supporting our hypothesis that the three genes may function redundantly.

### The CNS of homozygous *Ror^4^* mutant embryos resembles wild type

Next, we analyzed if the loss of *Ror* alone or of *Ror*, *otk* and *otk2* together had any effect on the development of the embryonic CNS. To that end we stained embryos homozygous mutant for the respective genotypes for the CNS axon marker BP102, for Fasciclin II, which marks a subset of CNS axons, and for Repo to visualize glial cells. After staining, fillet preparations of the CNS of stage 17 embryos were prepared.

**Fig. 4.**
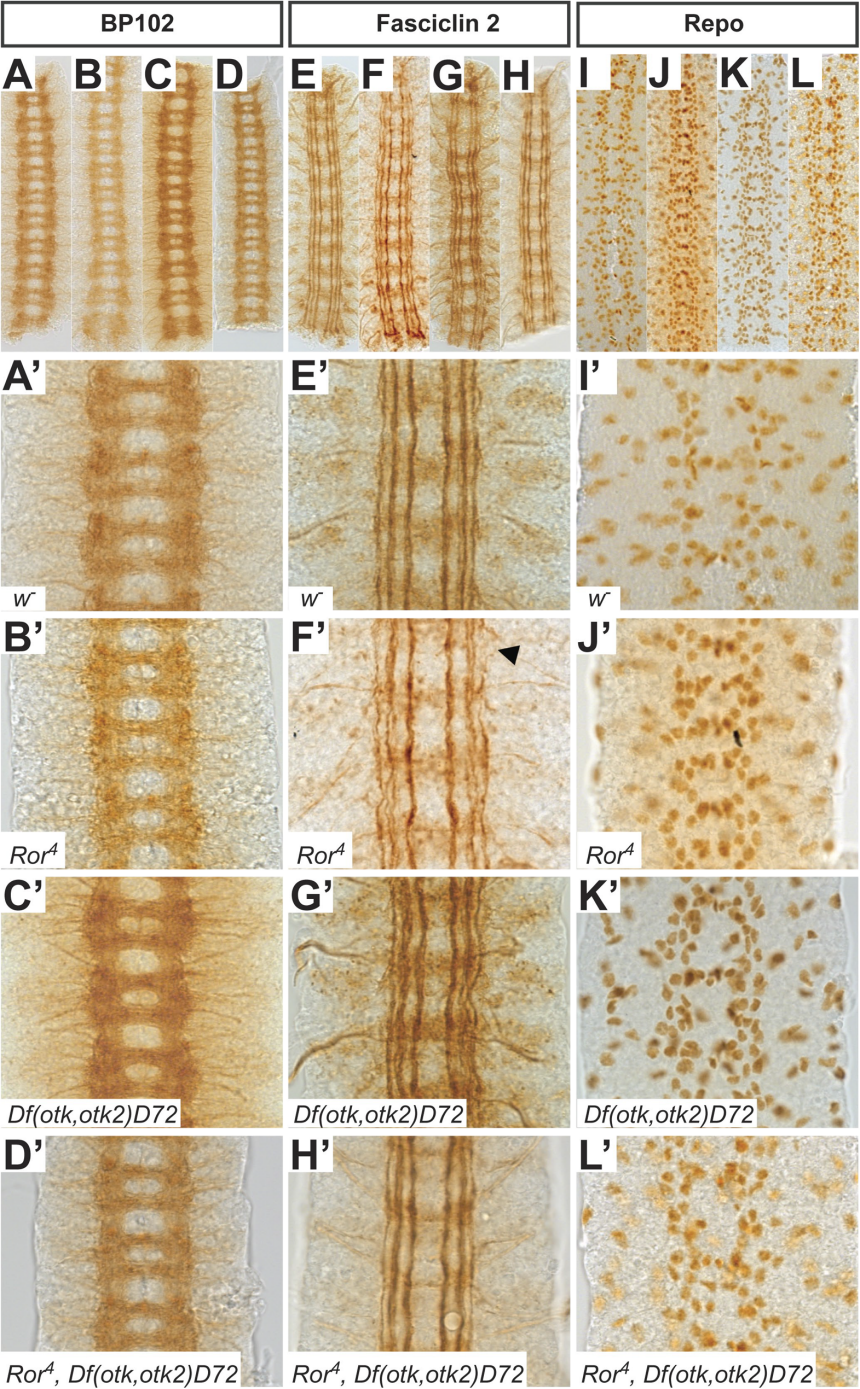
**Phenotypic analysis of the CNS in mutants for *Ror, otk* and *otk2*.** (A-D′) Axon tracts of the CNS are visualized using the BP102 antibody in *w^−^* (A), *Ror^4^* (B), *Df(otk,otk2)D72* (C) and *Ror^4^, Df(otk,otk2)D72* (D) embryos. All mutant embryos resemble the wild type. (E-H′) Fasciclin II labels the axons of a subset of neurons within the CNS. In *Ror^4^* mutant embryos (F,F′) occasional disruptions in the lateral fascicle are visible (arrowhead). In *otk*, *otk2* double mutants (G,G′) and *Ror*, *otk*, *otk2* triple mutants (H,H′) all fascicles look intact. (I-L′) Glial cells were visualized with the anti-Repo antibody. The pattern is not disturbed in any of the investigated mutants. Images (A′-L′) are higher magnifications of regions of the embryos shown in images (A-L). All images show three abdominal segments of late stage embryos. Anterior is up.

The CNS axon tracts visualized by BP102 in all analyzed mutant nervous systems resembled the wild type. The neuronal processes forming the longitudinal connectives were intact and the anterior and posterior commissures were present and separated from each other (Fig. 4A-D′).

In stage 16/17 embryos, Fasciclin II labels three longitudinal axon bundles, termed fascicles. In all genotypes we analyzed, all three fascicles were generally well formed and intact (Fig. 4E-H′). We rarely observed gaps in the lateral fascicles of *Ror^4^* mutant embryos (Fig. 4F′, arrowhead) at a frequency not significantly different from wild type. The pattern of glial cells was also not disturbed in any of the mutants analyzed (Fig. 4I-L′).

Taken together our results demonstrate that homozygous *Ror^4^* mutant embryos, *Df(otk,otk2)D72* double mutant embryos and *Ror^4^*, *Df(otk,otk2)D72* triple mutant embryos displayed a CNS phenotype resembling wild type.

### Adult *Ror^4^* mutant flies do not display obvious defects in planar cell polarity

In mice, the absence of Ror proteins leads to developmental defects associated with morphogenetic movements (Ho et al., 2012) and classical planar cell polarity (PCP) phenotypes in the cochlea (Yamamoto et al., 2008). Similar effects have been shown in other model organisms. In *Xenopus* for instance, Xror2 is required for convergent extension movements during embryogenesis (Hikasa et al., 2002). This indicates that PCP and convergent extension movements are regulated by Wnt signaling mediated through Ror proteins. While *Drosophila* Otk and Otk2 appear to have no function in establishing planar cell polarity (Linnemannstöns et al., 2014), their vertebrate homolog PTK7 was shown to control PCP signaling in several organisms including mouse, *Xenopus* and zebrafish (Hayes et al., 2013; Lu et al., 2004).

**Fig. 5.**
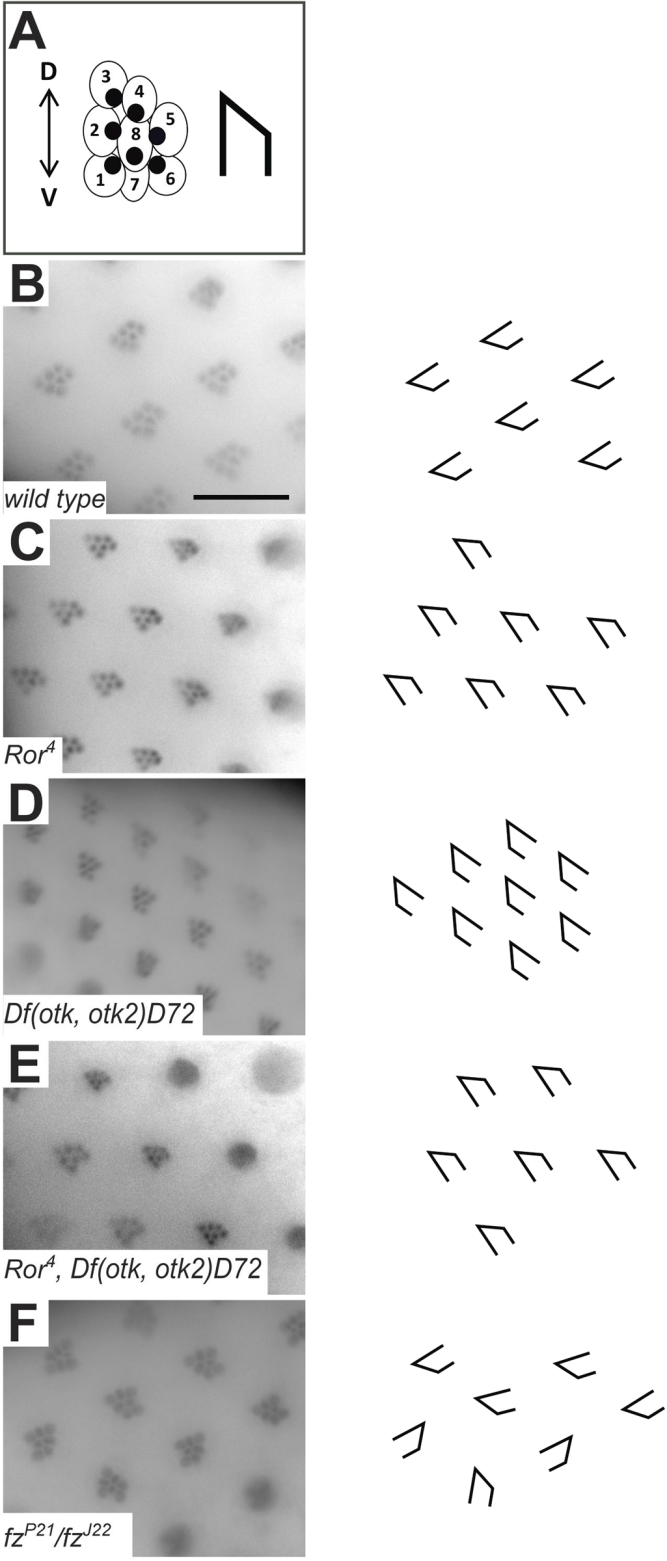
**Planar cell polarity in *Ror^4^* mutant eyes is not disturbed.** (A) Schematic representation of a *Drosophila* ommatidium. Each ommatidium is formed by eight photoreceptor cells. In a cross section only seven photoreceptor cells are visible, because the R7 and the R8 cell are positioned on top of each other. The visible cells resemble an arrowhead. (B) Wild-type ommatidia. (C) *Ror^4^* mutant eye. (D) *Otk* and *otk2* double mutant *Df(otk,otk2)D72*. (E) Triple mutant *Ror^4^,Df(otk,otk2)D72*. (F) *fz^J22^*/*fz^P2^* eye as positive control. The polarity of ommatidia is disturbed, all arrow-like shapes point in different directions. Scale bar: 20 µm.

To address whether *Drosophila* Ror plays a role in PCP signaling, we examined adult mutant flies for PCP defects. One of the planar polarized tissues in *Drosophila* is the composite eye. Each unit of the eye, called an ommatidium, is composed of eight photoreceptor cells: two inner and six outer photoreceptor cells. In sections, these cells resemble the shape of an arrowhead. All the ommatidia in the dorsal half of the eye point dorsally and in the ventral half they all point ventrally (Fig. 5A). When this D-V polarity is disturbed, the ommatidia are not oriented in the same direction anymore (Axelrod and McNeill, 2002; Zallen, 2007). The ommatidia in the eyes of homozygous *Ror^4^* mutant flies (Fig. 5C), *Df(otk,otk2)D72* double mutants (Fig. 5D) and *Ror^4^*, *Df(otk,otk2)D72* triple mutants (Fig. 5E) all formed arrow-like shapes that pointed in the same direction and therefore resembled the wild type (Fig. 5B), in contrast to the eyes of *fz* mutant flies, in which the ommatidia showed a randomized orientation (Fig. 5F).

**Fig. 6.**
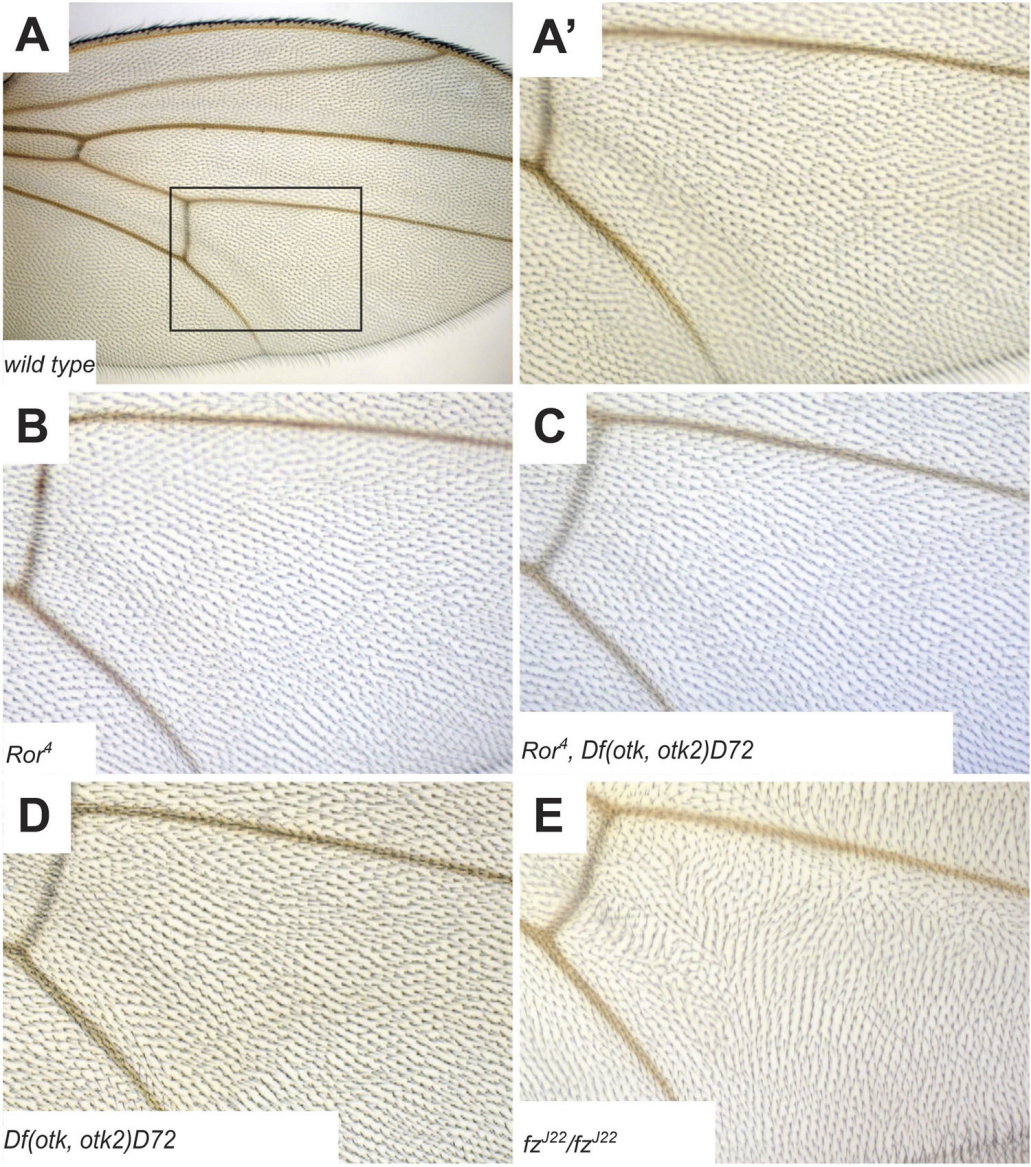
**Planar cell polarity in *Ror* mutant wings is normal.** (A) Overview of a wild-type *Drosophila* wing. (A′) Magnification of the boxed region in (A). (B) *Ror^4^* mutant wing. (C) Triple mutant *Ror^4^, Df(otk,otk2)D72*. (D) *otk, otk2* double mutant *Df(otk,otk2)D72*. (E) *fz^J22^*/*fz^J22^* wing as positive control showing disturbed orientation of wing hairs.

Another planar polarized tissue is the *Drosophila* wing. Here, the hairs secreted by every cell of the wing epithelium point distally. PCP defects can therefore be easily recognized by aberrant orientation of wing hairs (Axelrod and McNeill, 2002; Zallen, 2007). In *Ror^4^* mutant flies (Fig. 6B), *Df(otk,otk2)D72* double mutants (Fig. 6D) and *Ror^4^*, *Df(otk,otk2)D72* triple mutants (Fig. 6C), all wing hairs pointed in the same direction, in contrast to *fz^J22^*/*fz^J22^* mutant wings showing an aberrant orientation of wing hairs (Fig. 6E). Thus, in contrast to mammals, *Drosophila* Ror does not have an obvious function in the establishment of planar cell polarity.

### Analysis of genetic interactions between *Ror^4^* and other components of Wnt signal transduction

In a first approach to identify the pathway or biological process in which Ror functions, we analyzed genetic interactions of *Ror^4^* with additional components of Wnt signaling pathways. We generated allelic combinations of *Ror^4^* with mutant alleles for *fz*, *fz2*, *otk*, *otk2* and *Wnt5* to analyze their adult progeny for PCP phenotypes, fertility and viability. From each allelic combination we analyzed flies heterozygous for both mutant alleles, flies homozygous for *Ror^4^* and heterozygous for the second mutation, and flies homozygous for both. For *fz* and *fz2* we additionally analyzed *Ror^4^* in flies carrying two different alleles for *fz* or *fz2* in trans.

Homozygous *fz^J22^* flies are viable but display PCP defects in eyes, wings and body and the other two tested *fz* alleles are homozygous lethal. Transheterozygous flies for all three allele combinations are also viable with PCP defects (Table S1) (Jones et al., 1996). We did not detect any genetic interactions of *Ror^4^* with *fz* (Table S1). In the case of *fz2*, both alleles we tested are homozygous lethal. *fz2* transheterozygous animals are viable, display no PCP defects and are male and female sterile. For *fz2* and *Ror^4^* we did not observe any genetic interaction either (Table S1).

Flies homozygous mutant for *otk* and *otk2* single mutations are viable without any discernible phenotype, whereas double mutants for *otk* and *otk2* are male sterile (Linnemannstöns et al., 2014). Double mutants for *Ror^4^* and *otk* or *Ror^4^* and *otk2* were viable and fertile (Table S1). Flies homozygous mutant for all three genes were also male sterile and their CNS phenotype resembled wild type (Fig. 4D′,H′,L′).

Flies homozygous mutant for *Wnt5^400^* are viable but display CNS axon fasciculation defects (Fradkin et al., 2004). Animals homozygous mutant for *Wnt5^400^* and heterozygous for *Ror^4^* were viable, whereas animals homozygous for both *Wnt5^400^ and Ror^4^* were not obtained (Table S1), pointing to a functional interaction of both genes. However, flies transheterozygous for *Wnt5^400^* and *Ror^4^* with deficiencies for both genes or flies transheterozygous for the null alleles *Wnt5^400^* and *Wnt5^Gal4^* carrying *Ror^4^* over a deficiency were viable (Table S1). This finding could either point to allele-specific interactions between *Wnt5^400^* and *Ror^4^* or to the contribution of second site mutations on either the *Wnt5^400^* or the *Ror^4^* chromosome to the synthetic lethality of animals homozygous for *Wnt5^400^* and *Ror^4^*.

In conclusion, with the exception of animals homozygous mutant for both *Wnt5^400^* and *Ror^4^*, we did not observe any genetic interaction in terms of adult PCP phenotypes, sterility or lethality between *Ror* and *fz*, *fz2*, *otk, otk2* or *Wnt5*. However, we did observe strongly reduced embryonic viability of animals triply mutant for *Ror*, *otk* and *otk2*, pointing to redundant function of these three Wnt co-receptors.

### Ror binds specifically to the Wnt ligands Wg, Wnt4 and Wnt5

**Fig. 7.**
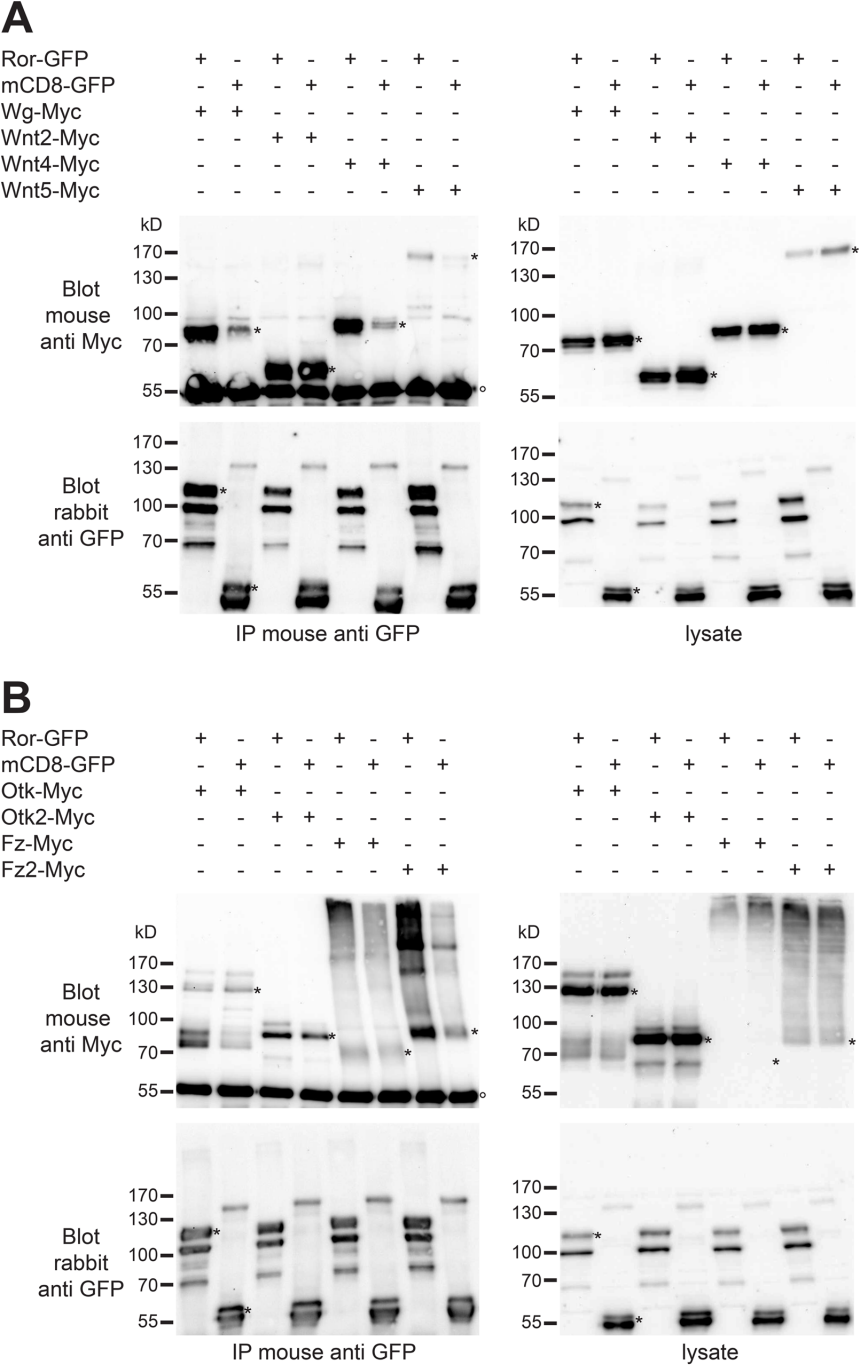
**Ror-GFP binds to Myc-tagged Wnts and Wnt receptors*.*** (A) The indicated constructs for Ror-GFP, CD8-GFP as negative control and Myc-tagged Wnts were transfected into *Drosophila* S2R+ cells. Co-immunoprecipitation from cell lysates was performed using mouse anti-GFP antibody followed by western blotting using mouse anti-Myc and rabbit anti-GFP antibodies. In the blot probed with anti-Myc antibody the denatured heavy chain of the immunoglobulin used in the IP is visible (marked with °). Bands representing the respective full-length proteins are marked with asterisks (*). (B) The indicated constructs for Ror-GFP, CD8-GFP as negative control and Myc-tagged Wnt receptors were co-transfected into *Drosophila* S2R+ cells. Co-immunoprecipitation from cell lysates was performed as above. IP, immunoprecipitation; Blot, western blot. Protein sizes are indicated in kilo Dalton (kD).

Vertebrate Ror proteins were shown to bind to several Wnt ligands and also to Fz receptors (Oishi et al., 2003). We therefore studied biochemical interactions of Ror with different Wnts by co-immunoprecipitation. To this aim, we co-overexpressed GFP-tagged Ror with Myc-tagged Wg, Wnt2, Wnt4 and Wnt5 under the control of the *actin5C* promoter in S2R+ cells. As negative control we co-transfected mCD8-GFP with the same Myc-tagged Wnts. The GFP-tagged proteins were immunoprecipitated from cell lysates using an anti-GFP antibody. Western blotting and detection with an anti-Myc antibody showed that Wg-Myc, Wnt4-Myc and Wnt5-Myc co-immunoprecipitated with Ror-GFP while co-IP with mCD8-GFP was much weaker, demonstrating the specificity of the interactions (Fig. 7A). For Wnt2-Myc, binding to Ror-GFP and CD8-GFP was of similar strength (Fig. 7A), questioning the specificity of this interaction.

### Ror binds to the Wnt receptor Fz2 and to the Wnt co-receptor Otk

In *Drosophila*, Fz and Fz2 constitute the core receptors for Wnt signaling (Bhanot et al., 1996, 1999). Otk and Otk2 have been shown recently to function as Wnt co-receptors (Linnemannstöns et al., 2014). To test whether Ror functions as an independent Wnt receptor, as a co-receptor together with Fz or Fz2, or may form receptor complexes together with Otk and Otk2, we performed co-immunoprecipitations with GFP-tagged Ror and Myc-tagged Fz, Fz2, Otk and Otk2 in S2R+ cells. All four receptors co-immunoprecipitated with Ror-GFP (Fig. 7B). For Fz-Myc and Otk2-Myc we also observed binding to CD8-GFP with similar strength as to Ror-GFP, questioning the specificity of their binding to Ror-GFP. For both Fz-Myc and Fz2-Myc the interpretation of co-IP experiments was hampered by the fact that these proteins had an unusual migration behavior in SDS-PAGE, being detectable mostly in a high molecular weight smear at the top of the lanes (Fig. 7B, top panels). However, IP of Fz-Myc and Fz2-Myc with anti Myc antibody revealed the presence of both proteins at the expected size in our lysates (Fig. S5B). Despite of these technical complications, our results are consistent with Ror functioning either as a Wnt co-receptor together with Fz2 and maybe also Fz, or by forming receptor complexes with Otk and maybe also Otk2.

### Ror-Myc overexpression does not lead to PCP defects in adult flies

For many regulators of PCP, it was shown that both their loss-of-function and their overexpression affected PCP (Adler et al., 1997; Krasnow and Adler, 1994). To test whether the overexpression of Ror-Myc has any influence on the establishment of PCP, we analyzed planar polarized tissues in adult flies ubiquitously overexpressing Ror-Myc. Overexpression of Ror-Myc was confirmed by western blot (Fig. S6A) and had no effect on embryonic survival (Fig. S6B). We did not observe any misorientation of ommatidia in the adult eye of Ror-Myc overexpressing flies (Fig. S7D). The wings of Ror-Myc overexpressing adult flies also displayed no PCP defect. All wing hairs pointed into the same direction: to the distal side of the wing (Fig. S8D), in contrast to the wings of flies mutant for *fz*, in which the hairs pointed in random directions (Fig. S8E).

### Overexpression of Ror-Myc does not affect nervous system development

**Fig. 8.**
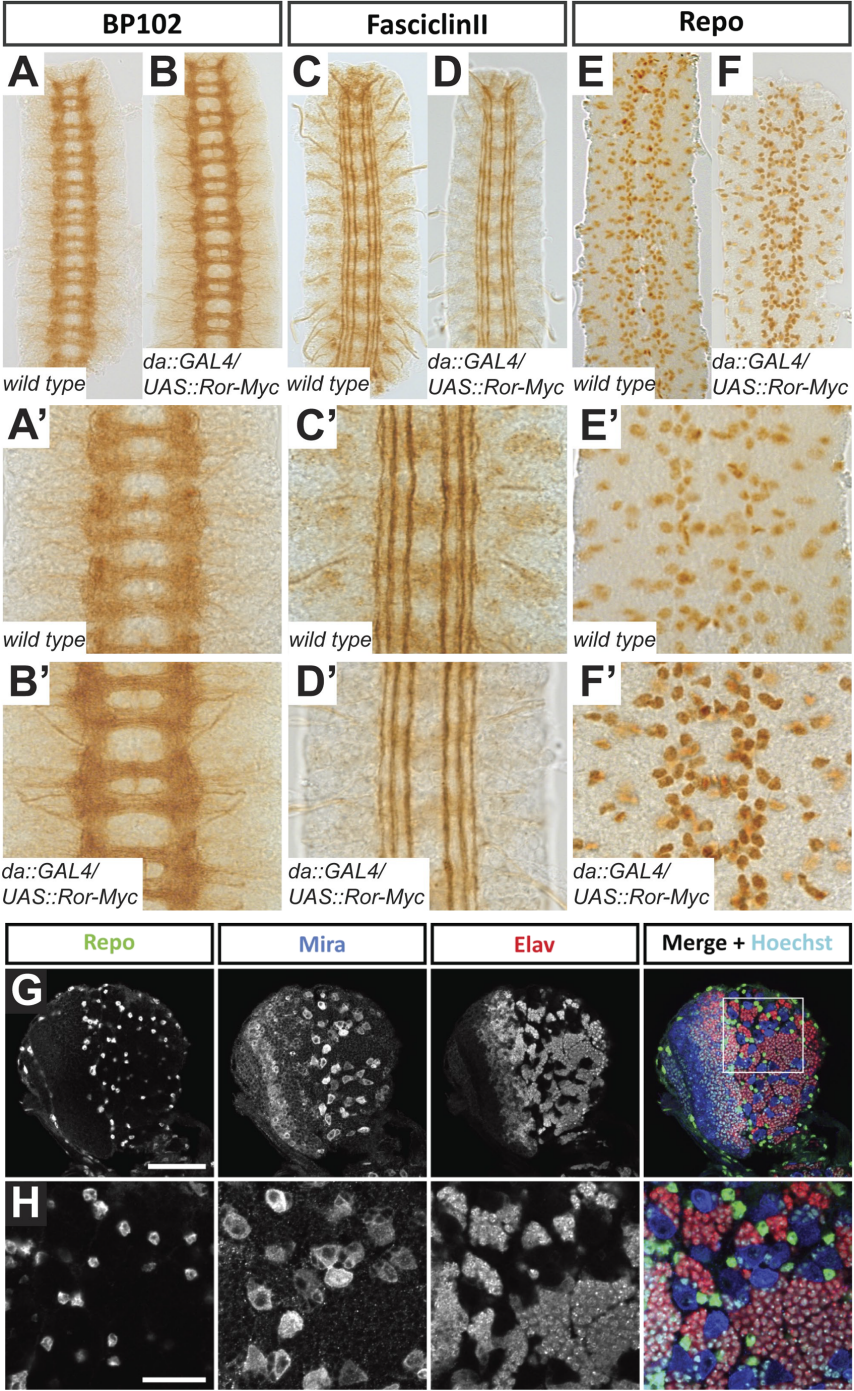
**The morphology of the ventral nerve cord in Ror-Myc overexpressing embryos is normal.** (A-B′) Axon tracts of the CNS visualized using the BP102 antibody in wild-type (A,A′) and Ror-Myc overexpressing embryos (B,B′). The CNS of embryos overexpressing *Ror* resembles the wild type. (C-D′) Longitudinal axon tracts visualized with Fasciclin II. In *da::Gal4/UAS::Ror-Myc* embryos (D,D′) all three fascicles are intact and resemble the wild type (C,C′). (E-F′) Glial cells visualized with the anti-Repo antibody. The pattern in *da::Gal4/UAS::Ror-Myc* embryos (F,F′) resembles the wild type (E,E′). Images (A′-F′) are higher magnifications of the preparations shown in images (A-F). All images show three abdominal segments of late stage embryos; anterior is up. (G,H) Confocal images of the CNS of a third instar *da::Gal4/UAS::Ror-Myc* larva. Expression patterns of the indicated proteins are normal and resemble the wild type (cf. Fig. 2, Fig. S2). (G) Overview of a brain hemisphere. (H) Higher magnification of the larval brain lobe. Scale bars: G, 50 µm; H, 20 µm.

To assess whether ubiquitous overexpression of Ror-Myc affects development of the nervous system, we stained embryos of the genotype *da::GAL4/UAS::Ror-Myc* with a variety of nervous system-specific antibodies. Staining with the BP102 antibody, which visualizes all CNS axons, revealed the typical ladder-like axon pattern of the CNS both in Ror-Myc overexpressing embryos and in the wild-type control (Fig. 8A-B′). In each CNS segment, two clearly separated commissures were visible and all segments were connected by the longitudinal connectives (Fig. 8A-B′). In a staining for Fasciclin II, the *da::GAL4/UAS::Ror-Myc* embryos also resembled the wild-type control. Three parallel axon bundles were observed on either side of the midline, the lateral, the intermediate and the medial fascicle (Fig. 8C-D′). We did not observe any breaks in the fascicles, crossings at the midline or any other defects (Fig. 8D,D′). The glial cell pattern of embryos overexpressing Ror-Myc visualized by staining against Repo was not altered and comparable to the wild type (Fig. 8E-F′). We did not notice any missing or misplaced glia (Fig. 8F,F′).

To assess a potential neural phenotype at later stages of development, we examined the CNS of *da::GAL4/UAS::Ror-Myc* larvae. We stained brains of third instar larvae with the neuroblast marker Miranda (Mira), the neuronal marker Elav and the glial marker Repo. In all analyzed brains, the morphology and size of the brain were not affected. The staining showed normal patterns of neuroblasts, neurons and glia cells in the brain (Fig. 8G,H).

## DISCUSSION

Ror kinases have multiple functions during vertebrate and *C. elegans* development (Endo and Minami, 2017). However, nothing is known so far about the function of Ror and Nrk, the two *Drosophila* homologs of Ror. Here we present the first functional analysis of *Drosophila* Ror. We describe in detail the expression pattern and subcellular localization of the *Drosophila* Ror protein, present the phenotypic analysis of a null mutant allele of *Ror* and analyze the phenotypic consequences of Ror overexpression. In addition, we investigate the binding of Ror to several Wnt ligands and Wnt receptors.

To investigate the expression pattern and subcellular localization of Ror, we generated a transgenic line carrying a BAC in which GFP was fused to the C-terminus of Ror by recombineering in bacteria. The expression pattern of the Ror protein matched the published pattern of the Ror mRNA (Wilson et al., 1993) and confirmed that Ror is predominantly expressed in the nervous system and in somatic muscles. As expected for a single-pass transmembrane protein, Ror labels the plasma membrane of neurons. We detected Ror on the membrane of perikarya and neurites, consistent with a potential function in axon pathfinding or fasciculation.

Animals homozygous mutant for the null allele *Ror^4^* were viable and fertile and resembled wild type in every phenotype we analyzed. To identify proteins that might function redundantly with Ror and thus explain the lack of a mutant phenotype, candidate genes were checked for genetic interactions with *Ror^4^*. However, with the exception of animals doubly homozygous for *Wnt5^400^* and *Ror^4^*, we did not observe any allelic combination that was lethal due to the absence of *Ror* function. With respect to embryonic viability, we did observe genetic interaction between *Ror*, *Otk* and *Otk2*. Embryos triply homozygous mutant for all three genes showed strongly reduced embryonic survival, pointing to redundant function of these Wnt co-receptors.

In vertebrates, Ror2 regulates planar cell polarity of epithelial tissues and is required for proper morphogenesis (Hikasa et al., 2002; Schambony and Wedlich, 2007; Yamamoto et al., 2008). By contrast, we did not observe any defects in PCP of wings or eyes upon mutation or overexpression of *Drosophila* Ror. In the wing, the lack of a phenotype is consistent with the lack of expression of Ror in the wing imaginal disc epithelium. Lack of PCP phenotypes was also observed for mutations in *otk* and *otk2*, the two fly orthologs of vertebrate PTK7 (Linnemannstöns et al., 2014), although PTK7 is required for PCP in vertebrates (Berger et al., 2017; Paudyal et al., 2010; Shnitsar and Borchers, 2008; Yen et al., 2009). The reason for the dispensability of Ror, Otk and Otk2 for the regulation of ‘classical’ PCP phenotypes in flies may lie in the different cell biological processes that are regulated by PCP genes in vertebrates and flies. While PCP in vertebrates regulates in most cases cell movement, e.g. convergent extension in gastrulation, PCP in flies regulates the formation of subcellular structures, e.g. of cuticular hairs in the wing epithelium or the orientation of small groups of cells in an epithelium, like bristle sense organs in the notum.

Genetic and biochemical evidence has been provided that vertebrate Ror2 transduces the signal of the non-canonical Wnt ligands Wnt5a and Wnt11 (Bai et al., 2014; Cerpa et al., 2015; Maeda et al., 2012; Oishi et al., 2003; Schambony and Wedlich, 2007) and can bind to the canonical ligands Wnt1 and Wnt3 (Billiard et al., 2005; Li et al., 2008). Vertebrate Ror2 has also been shown to form complexes with several additional Wnt receptors, including Fzd family members, PTK7 and Vangl2 (Gao et al., 2011; Martinez et al., 2015; Podleschny et al., 2015; Yamamoto et al., 2008). We show here that *Drosophila* Ror is engaged in protein complexes together with the Wnt ligands Wg, Wnt4 and Wnt5 and with the Wnt receptors Fz2 and Otk, strongly indicating that *Drosophila* Ror also functions as a Wnt receptor.

Together, our findings provide the basis for detailed analyses on the function of Ror in *Drosophila* nervous system development. It will be of particular interest to study the contribution of Ror to Wnt5 signaling and to uncover potential redundancies with additional Wnt receptors expressed in the nervous system, including Otk, Otk2, Nrk and Ryk.

## MATERIALS AND METHODS

### Fly stocks and genetics

The following stocks were used in this study: *wg^CX4^ b^1^ pr^1^/CyO* (#2980), *Wnt4^C1^/CyO* (#6651), *Wnt2^L^/CyO* (#6909), *Wnt5^Gal4^* (#59034), *Df(1)N19/FM6* (#970), *Df(2L)ED729/SM6a* (#24134) *daughterless::Gal4* (#5460) (Bloomington *Drosophila* Stock Center, Bloomington, USA; stock numbers given in parentheses). *P(GSV3)GS8107* (#201394, Kyoto *Drosophila* Stock Center); *fz^J22^*, *fz^P21^* (gifts from Paul Adler, University of Virginia, Charlottesville, USA), *fz^R52^* (gift from Ken Cadigan, University of Michigan, Ann Arbor, USA); *fz2^C2^* (gift from G. Struhl, Columbia University, New York City, USA); *Df(3L)469-2* (Bhanot et al., 1999)*;* Wnt5^400^ (Fradkin et al., 2004); *otk^A1^, otk2^C26^, Df(otk,otk2)*D72 (Linnemannstöns et al., 2014); Transgenic fly lines for the constructs UAS::Ror-Myc and Ror::Ror-eGFP were generated as described (Bischof et al., 2007; Fish et al., 2007).

### Molecular biology

The coding regions of full-length *Ror*, *otk*, *otk2*, *fz1, fz2*, *wg*, *Wnt2, Wnt4, Wnt5* and *CD8* were amplified and the PCR products cloned into pENTR vector using the pENTR Directional TOPO Cloning Kit (Invitrogen). For expression in S2r+ cells and for generation of transgenic flies, constructs were recombined into different expression vectors (pAWG, pAWM, pPWG-attB; Murphy lab, Carnegie Institution of Washington, Baltimore, USA) using Gateway technology (Invitrogen). A BAC construct encoding a C-terminal Ror-eGFP fusion protein under control of its endogenous promoter was generated from the BAC CH322-82M14 according to the protocol described in (Venken et al., 2008).

### Immunohistochemistry and antibodies

Embryos were collected at 25°C and dechorionated in bleach for 5 min. Embryos were washed with water, transferred into a 1:1 mixture of heptane and 4% formaldehyde in PBS buffer and were then fixed by rocking for 20 min. Formaldehyde and PBS were removed and methanol was added to form a 1:1 mixture of heptane and methanol. Embryos were then devitellinized by vigorous shaking for 30 s. After removing most of the liquid, devitellinized embryos were washed three times with methanol and either stored at −20°C in methanol or directly processed for staining. For adult larval brains, flies were reared at 25°C, dissected in phosphate buffered saline (PBS; pH 7.4) and fixed in 4% formaldehyde/PBS for 20 min. Fixed samples were washed three times in 0.1% Triton X-100/PBS (PBT) for 20 min and were then blocked in 5% Normal Horse Serum (NHS)/1% Triton X-100/PBS for 30 min. Blocked brains were rinsed three times in PBS to remove excess Triton X-100 and then incubated overnight at 4°C in 5% NHS/PBT with primary antibodies. Embryos were blocked in 5% NHS/PBT for 30 min and then incubated overnight at 4°C in 5% NHS/PBT with primary antibodies. Samples were washed three times for 20 min in PBT at room temperature. Samples were blocked with 5% NHS/PBT for 30 min and incubated with secondary antibodies and DAPI in 5% NHS/PBT at 1:500 for 2 h at room temperature. Secondary antibody solution was removed and samples were washed three times for 20 min with PBT. Samples stained with fluorophor-conjugated secondary antibodies were mounted in Vectashield (Vector Laboratories, Burlingame, USA) or Mowiol. Confocal microscopy was performed using Zeiss LSM510 Meta and Zeiss LSM880 Airyscan confocal microscopes. Light sheet microscopy was performed using a Zeiss Lightsheet Z1 microscope (kindly provided by Carl Zeiss). For flat preparations of the embryonic CNS, biotinylated secondary antibodies were used in combination with the Vectastain ABC kit (Vector Laboratories). For dissection, peroxidase stained embryos were transferred onto a glass slide. The anterior and the posterior part of the embryos were cut off with the tip of a 26 G needle and afterwards the dorsal side of the embryos was sliced open. Next, the lateral sides of the embryo were hinged to the side and the gut was removed. Finally, the stained and dissected nervous systems were transferred onto a fresh glass slide, mounted in 10 µl 50% glycerol and covered with a 24×50 mm coverslip, which was fixed on the slide with nail polish. For preparation of larval muscles, L3 larvae were washed in PBS to remove residual food and placed on a plate with PBS. Larvae were fixed with needles and cut open between the lateral trunks of the trachea along the a/p-centerline. Larval cuticle was nicked at the anterior and posterior end to the lateral side and the cuticle was unfolded to fix it with needles onto the plate to flatten it. Inner organs were removed, imaginal discs remained. Torsos were washed with PBS to remove residual tissue and then shortly once with 3.7% formaldehyde in PBS and then fixed for 15 min in 3.7% FA. Fixative was removed and the torso washed three times with PBS. After the needles were removed the torsos were transferred to a tube and washed three times with PTX (0.1% Triton X-100 in PBS) for 10 min before immunostaining.

Images were processed using Photoshop CS5 (Adobe) and assembled using Illustrator CS5 (Adobe).

The following primary antibodies were used: guinea pig anti Miranda 1:1000 (Kim et al., 2009); guinea pig anti Otk, 1:1000 (Linnemannstöns et al., 2014); rabbit anti Otk2, affinity-purified, 1:100 (Linnemannstöns et al., 2014); mouse and rabbit anti GFP, 1:1000 (A11120 and A11121, Invitrogen); mouse BP 102, 1:50; mouse anti Elav 9F8A9, 1:30; mouse anti Repo 8D12, 1:20; mouse anti Fasciclin 2 1D4, 1:20; mouse anti Dlg 4F3, 1:20 (DSHB, University of Iowa, USA); Alexa Fluor^®^ 647 AffiniPure Goat Anti-Horseradish Peroxidase (Jackson ImmunoResearch).

Secondary antibodies conjugated to Cy2, Cy3 (Jackson ImmunoResearch Europe, Newmarket, UK), Alexa Fluor 647 (Invitrogen) and biotin (Vector Laboratories) were used at 1:400 dilution. DNA was stained with 4′,6-Diamidino-2-Phenylindole (DAPI; Invitrogen).

### Mounting of adult wings

Wings were removed from adult flies and dehydrated in 100% isopropanol for at least 5 min. Wings were placed on a glass slide and the isopropanol was allowed to evaporate. Wings were mounted with a small drop of Roti^®^ Histokitt (Roth) for microscopic analysis.

### Analysis of PCP defects in adult *Drosophila* eyes

To analyze adult eyes for defects in planar cell polarity, the heads of anaesthetized flies were cut off and immediately mounted in immersion oil (Zeiss) on a glass slide. To protect the heads from being squashed, the glass slide used for mounting had coverslips glued onto two sides to increase the space between slide and coverslip. The polarity of the photoreceptor cells was then analyzed by light microscopy.

### Viability measurements

Viability was determined by aligning 100 embryos on apple juice agar plates. The embryos were allowed to develop at 25°C and hatching rates were recorded after at least 24 h. All experiments were done in triplicate. The statistical significance of the results was calculated using the independent samples Student's *t*-test.

### Culturing and transfection of *Drosophila* Schneider S2R+ cells

For cell culture experiments the Schneider cell line S2R+ was used (Schneider, 1972). Cells were maintained at 25°C in *Drosophila* S2 medium (Gibco), supplemented with 10% FCS (fetal calf serum), 50 U/ml penicillin, 50 µg/ml streptomycin. For each (co-)transfection, two wells with 2 million cells each were transfected with 4 µg plasmid DNA using FuGENE® HD transfection reagent (Promega, Madison, USA). The cells were incubated at 25°C for 96 h before harvesting.

### Western blots and co-immunoprecipitation (Co-IP)

Protein extraction and western blots were performed according to standard procedures (Wodarz, 2008). For Co-IPs, transiently transfected S2R+ cells were lysed in 1 ml cold Co-IP lysis buffer (50 mM Tris-Cl pH 7.5, 150 mM NaCl, 1% Triton X-100 with protease inhibitors) by pipetting up and down in a 1000 µl micropipette and incubation on ice for 30 min. The lysates were centrifuged and the supernatant was transferred into fresh tubes and pre-cleared with Protein G Sepharose beads (BioVision, Milpitas, USA) for 1 h on a rotator at 4°C. After pre-clearing, 20 μl of each sample were kept as input control. Lysate corresponding to 1 mg of total protein was incubated with 2 µg of mouse anti GFP (Invitrogen, A11120) for 1 h at 4°C. Next, 20 µl Protein G Sepharose beads were added to the lysates and incubated overnight at 4°C on a rotator. Beads were washed three times with 1 ml Co-IP buffer. After removal of all remaining liquid with a syringe, the beads were boiled with 2× SDS-loading buffer and stored at −20°C or used directly for SDS-PAGE and western blot.

Antibodies used for western blot were rabbit anti GFP, 1:2000 (A11122, Invitrogen) and mouse anti c-myc (9E10), 1:200 (DSHB).

## Supplementary Material

Supplementary information
