# Supplementary material for: Drosophila Ror is a nervous system-specific co-receptor for Wnt ligands
Source: Biol Open. 2018 Oct 19;7(11):bio033001. doi: 10.1242/bio.033001 (PMC6262871; doi:10.1242/bio.033001)
Supplement: Supplementary information [file biolopen-7-033001-s1.pdf]

Supplemental Material

Supplemental Figures

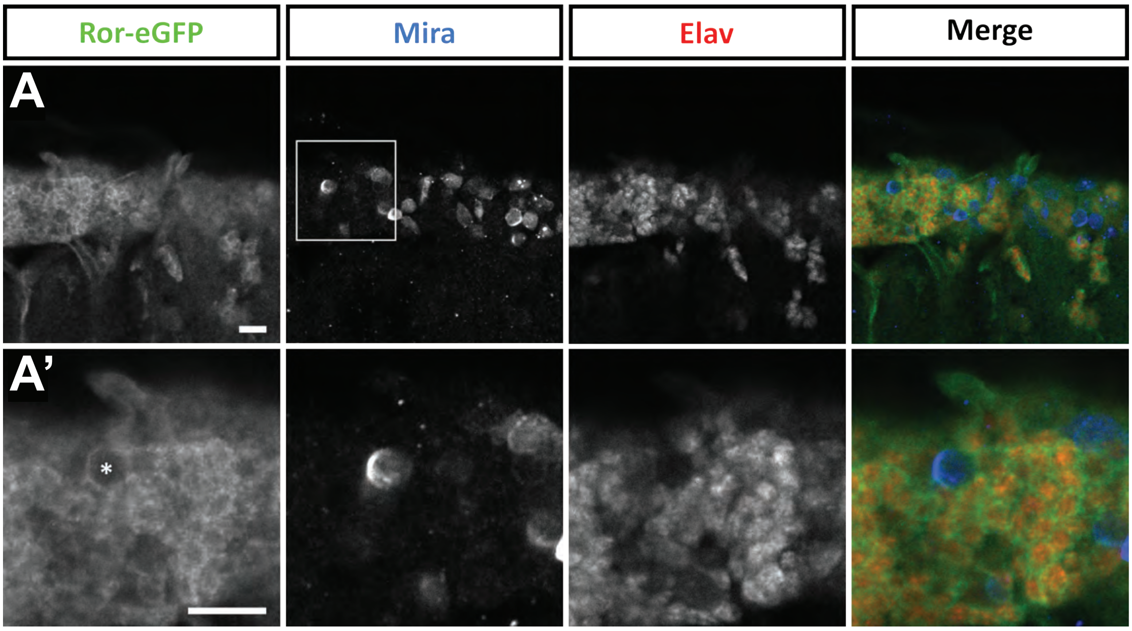

Figure S1, related to Fig. 1. Ror-eGFP is expressed in embryonic neuroblasts and neurons. (A) Ventral nerve cord of a stage 16 Ror-eGFP embryo stained against GFP (green in merged image), Miranda (blue in merged image) and Elav (red in merged image). Ror-eGFP is expressed in the membrane of Elav-expressing neurons. (A’) Region corresponding to the box in (A). At higher magnification Ror-eGFP is detectable in embryonic neuroblasts (asterisk). Scale bars = 10 µm.

Biology Open • Supplementary information

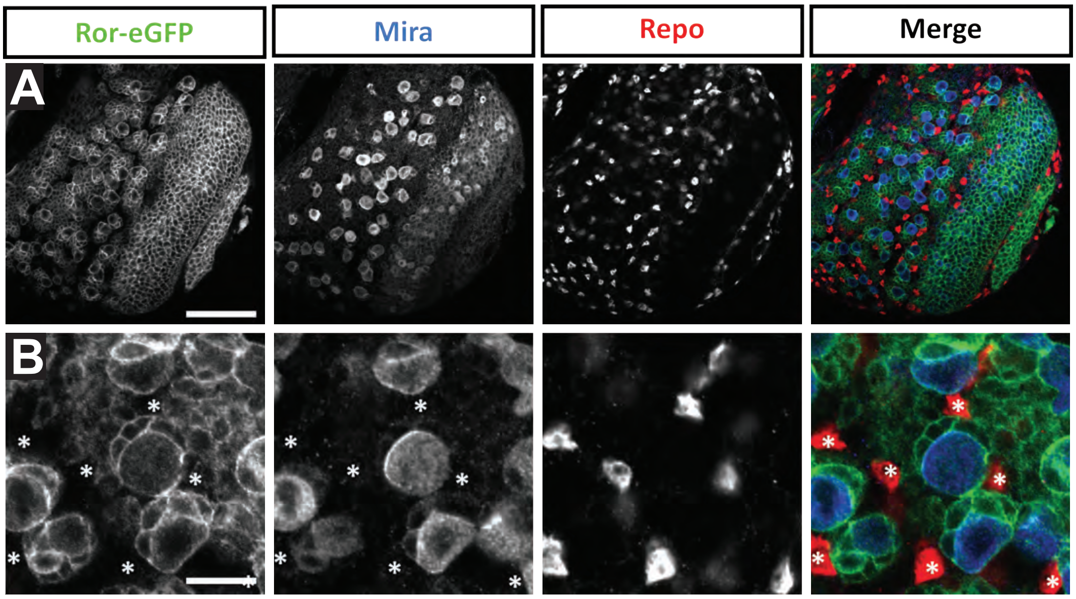

Figure S2, related to Fig. S2. Ror-eGFP is not expressed in glial cells within the central nervous system of third instar larvae. (A) Overview of a larval brain lobe stained against GFP (green in merged image), Miranda (blue in merged image) and Repo (red in merged image). (B) Higher magnification of the larval brain lobe. Note that no Ror-eGFP signal is detectable in the membrane of Repo-positive glia cells (asterisks). Scale bars: A = 50 µm; B = 10 µm.

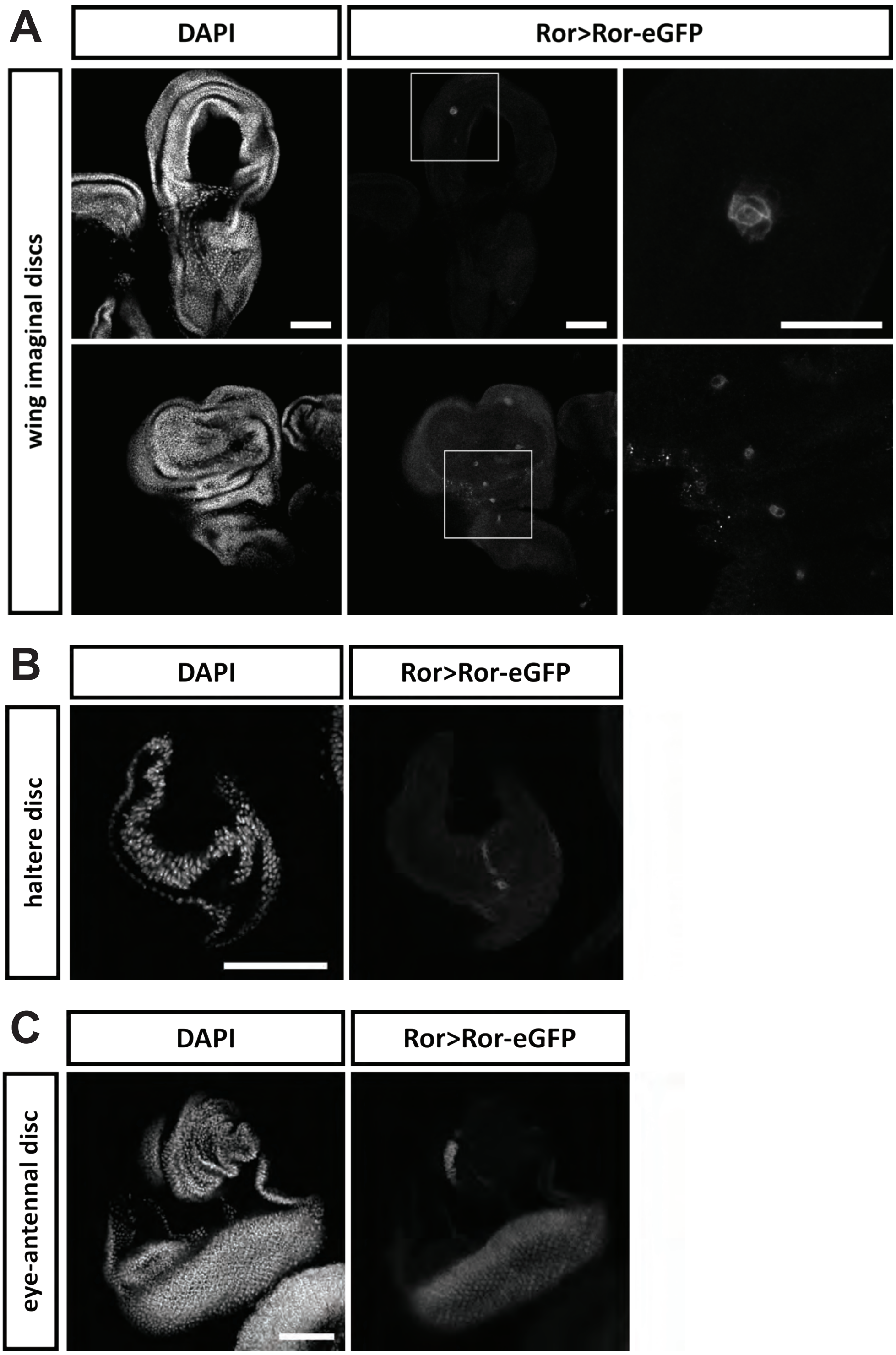

Biology Open • Supplementary information

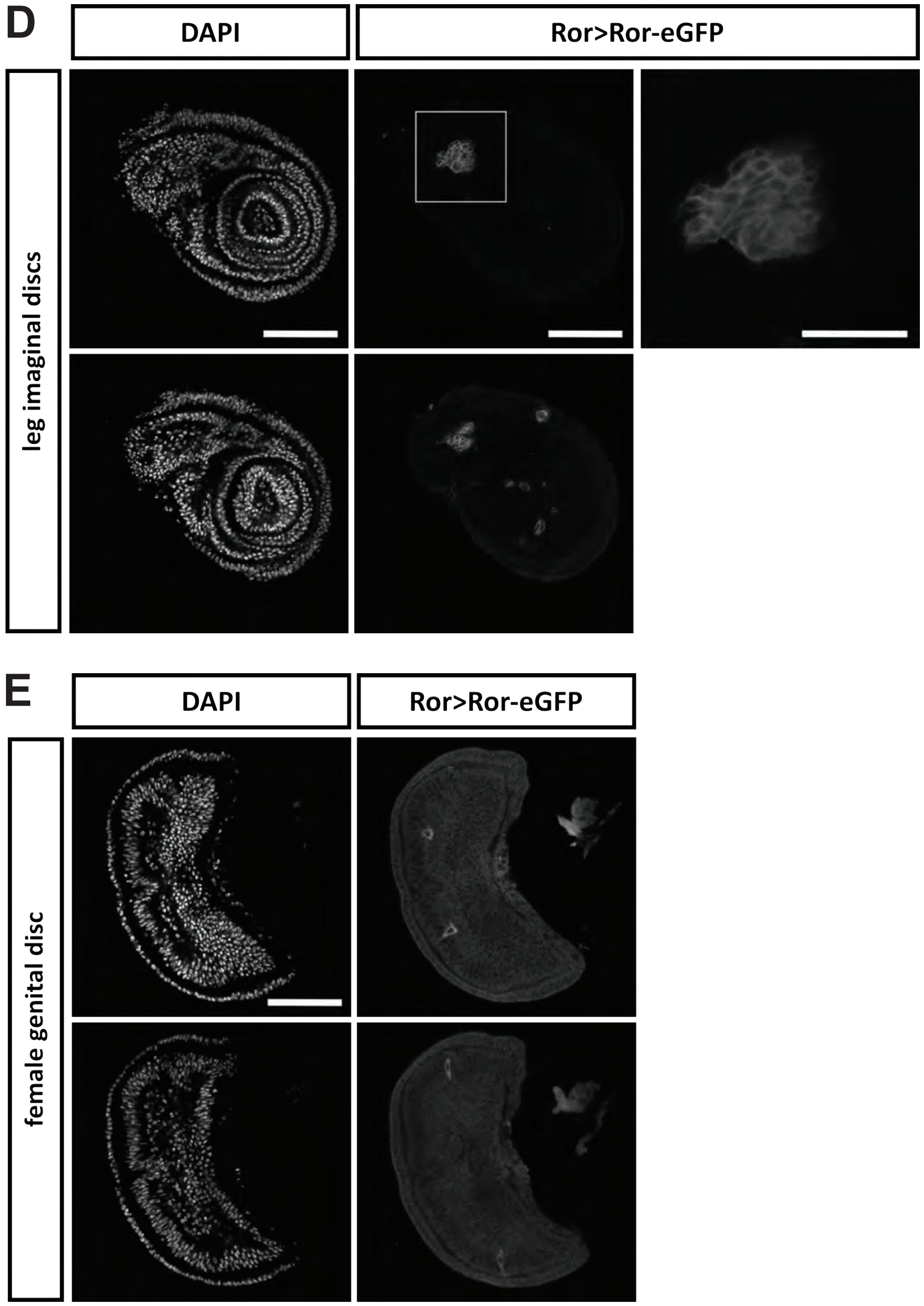

Biology Open • Supplementary information

Figure S3, related to Fig. 2. Ror-eGFP expression in third instar larval imaginal discs. (A) Wing imaginal discs. Two different discs are shown. The images were taken at different focal planes to visualize the different types of Ror-eGFP positive cell clusters. The boxed regions are shown at higher magnification in the right panels. (B) Haltere imaginal disc. (C) Eye-antennal imaginal disc. The eye part with Ror-eGFP positive photoreceptor cells is at the bottom, the antennal part with a single cluster of Ror-eGFP positive cells is at the top of the panel. (D) Leg imaginal disc, two focal planes of the same imaginal disc are shown. The boxed region is shown at higher magnification in the top right panel. (E) Female genital disc, two focal planes of the same imaginal disc are shown. Scale bars = 50 µm; Scale bars in magnifications of boxed regions in (A) and (D) = 20 µm.

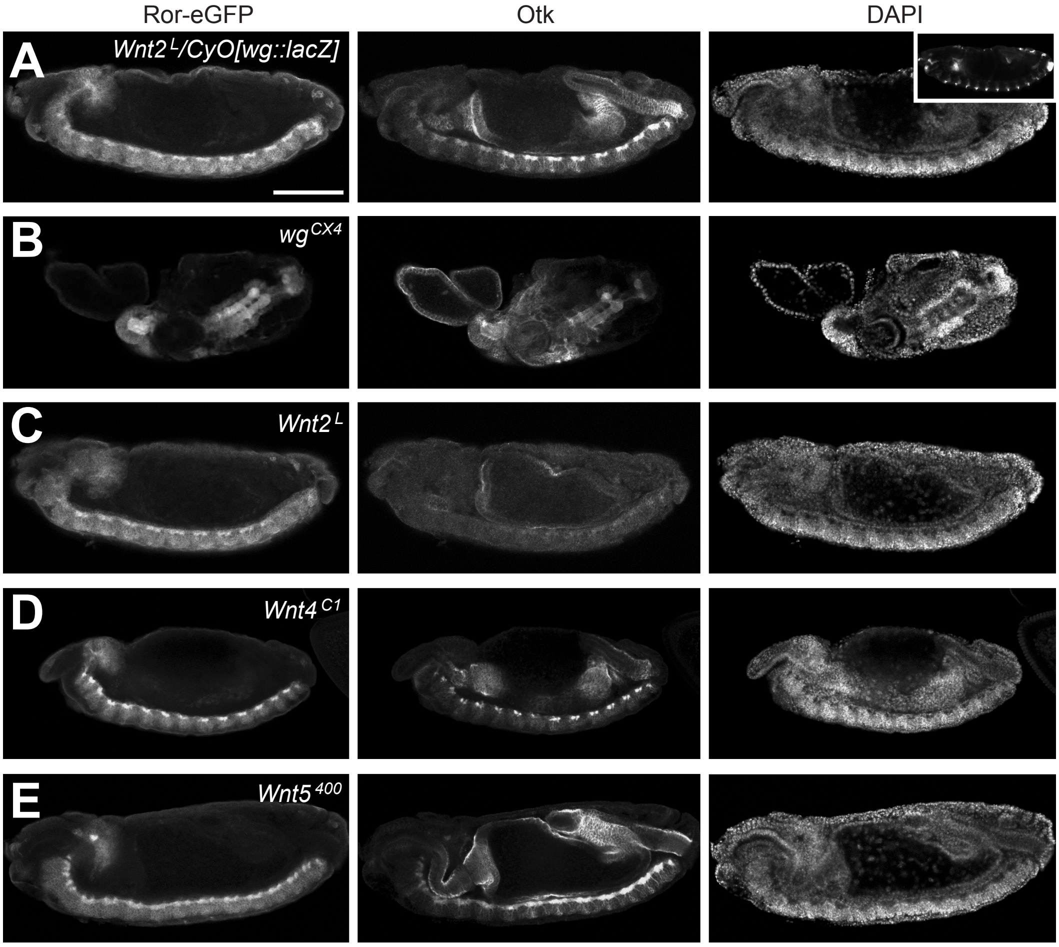

Figure S4, related to Fig. 1. Embryonic expression of Ror-eGFP is not affected by mutations in Wnt genes. Embryos were stained for GFP, Otk, DAPI and β-galactosidase (only shown in inset of the right panel of [A]), which labels the balancer chromosome in heterozygous *Wnt* mutants. (A) *Wnt2^{L}/CyO[wg::lacZ]* heterozygous embryo at stage 16. (B) Homozygous *wg^{CX4}* mutant embryo. Note that the exact developmental stage of the embryo cannot be determined due to the strong morphogenesis defects. (C) Homozygous *Wnt2^{L}* mutant embryo, stage 15. Note reduced expression of Otk in the *Wnt2^{L}* mutant embryo compared to the heterozygous embryo in (A) and all other genotypes shown. (D) Homozygous *Wnt4^{C1}* mutant embryo, stage 14. (E) Homozygous *Wnt5^{400}* mutant embryo, stage 15. Anterior is to the left, dorsal is up. Scale bar = 100 µm.

Biology Open • Supplementary information

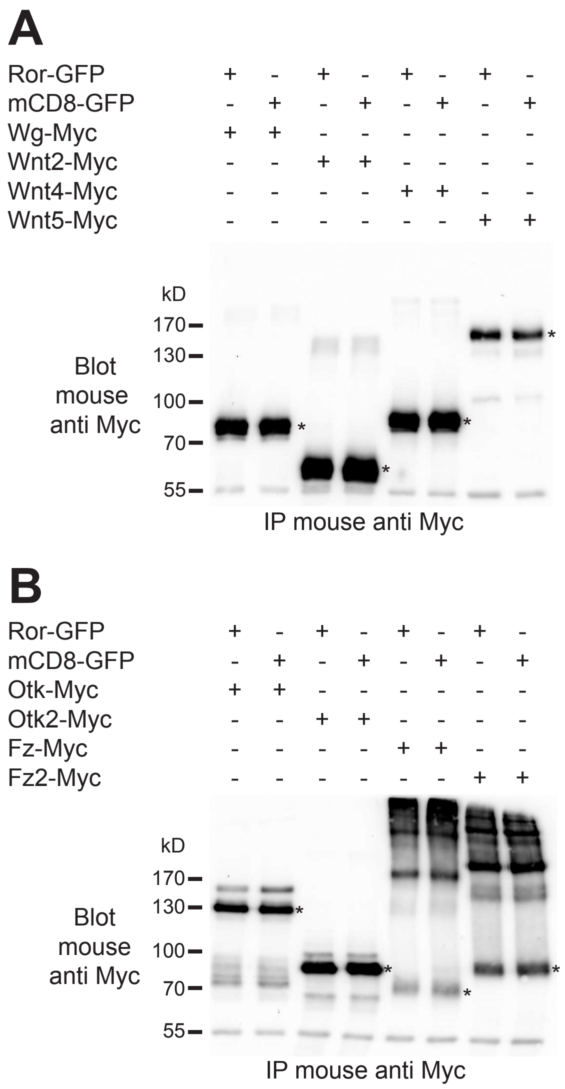

<u>Figure S5, related to Fig. 7.</u> IP of Myc-tagged Wnts and Wnt receptors from S2R+ cell lysates. Lysates of S2R+ cells transfected with the indicated constructs were subjected to IP with mouse anti c-Myc monoclonal antibody 9E10. Blots were probed with the same antibody as used for IP. (A) S2 cells cotransfected with constructs encoding Ror-GFP or CD8-GFP and Myc-tagged Wnt proteins. (B) S2 cells cotransfected with constructs encoding Ror-GFP or CD8-GFP and Myc-tagged Wnt receptors. Bands corresponding to full-length proteins are marked by asterisks (*). Note the abnormal migration behavior of Fz-Myc and Fz2-Myc which are mostly detectable as a high molecular weight smear.

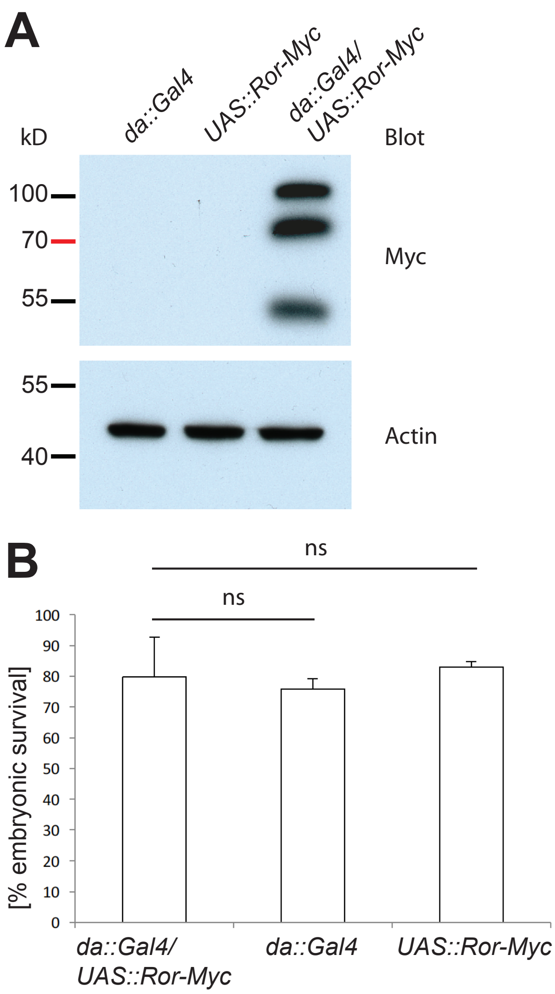

Figure S6, related to Fig. 8. Ror-Myc overexpression via the UAS-Gal4 system. (A) Whole embryo protein lysates of the indicated genotypes were subjected to SDS-PAGE and Western Blotting. The signal for the Ror-Myc fusion protein is clearly visible in the sample from embryos carrying both the Gal4 driver and the UAS-Ror-Myc transgene. (B) The hatching rate of embryos ubiquitously overexpressing Ror-Myc was not significantly different from *da*::*Gal4* and *UAS::Ror-Myc* embryos. n = 100. Data were obtained by repeating each experiment at least three times. The error bars represent the standard deviation of the mean. ns, p-value > 0.05 (independent samples Students t-test).

Biology Open • Supplementary information

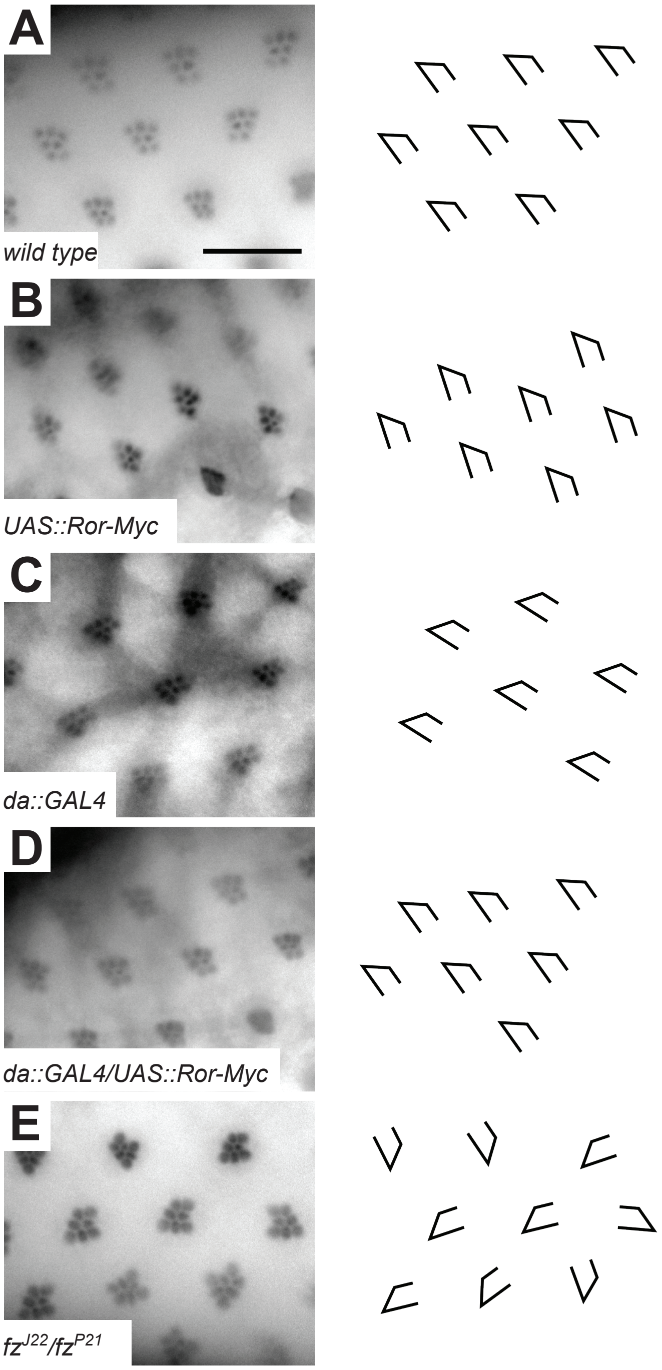

Figure S7, related to Fig. 8. Ror-Myc overexpression does not affect PCP in the eye. (A) Wild type ommatidia. (B) *UAS::Ror-Myc* negative control. (C) *da::Gal4* negative control. (D) Eye of an adult Ror-Myc overexpressing fly. All ommatidia point in the same direction. (E) *fz*^{J22}/*fz*^{P21} eye as positive control. Ommatidia are oriented randomly. Scale bar = 20µm.

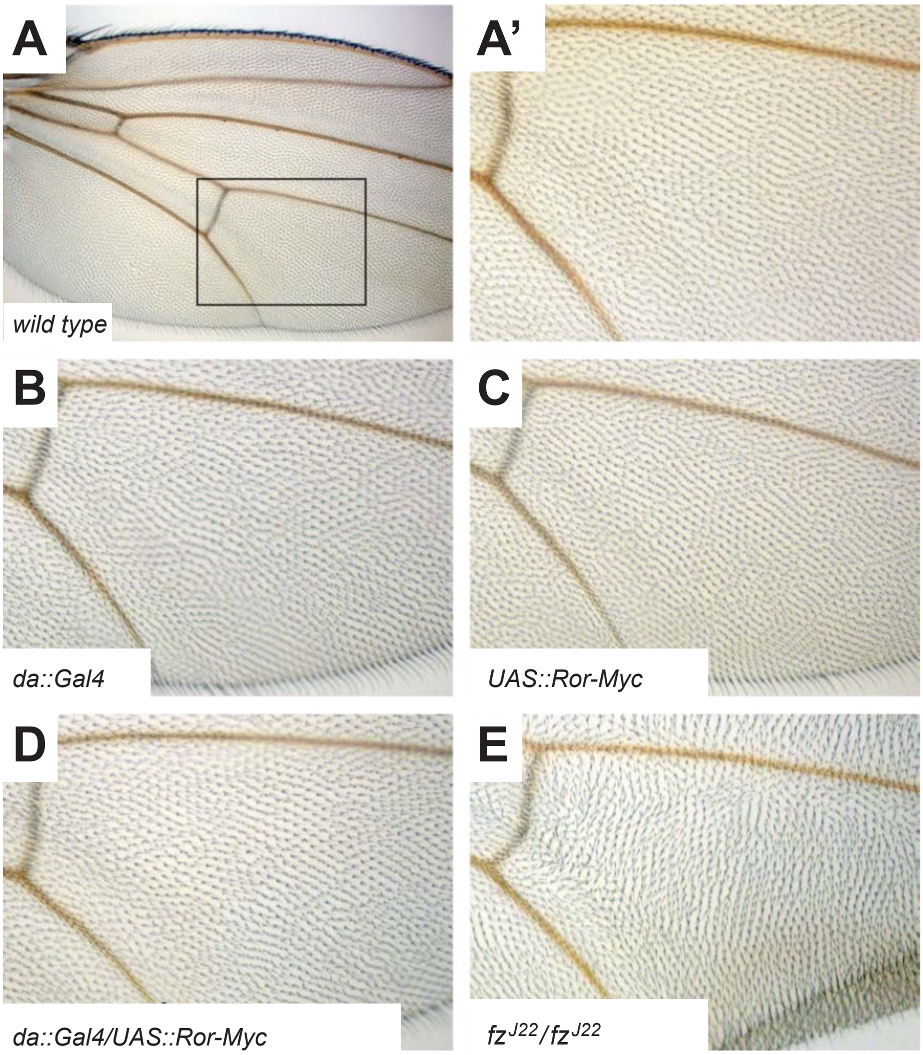

Figure S8, related to Fig. 8. Ror-Myc overexpression does not affect PCP in the wing. (A) Overview of a wild type *Drosophila* wing. (A') Magnification of the boxed region in (A). (B) *da::Gal4* control. (C) *UAS::Ror-Myc* control. (D) Wing of a fly overexpressing Ror-Myc under control of *da::Gal4*. (E) fz^{J22}/fz^{J22} wing as positive control showing disturbed orientation of wing hairs.

Biology Open • Supplementary information

Table S1: Ror genetically interacts with Wnt5. Genetic interactions of the *Ror*^{4} allele with three *fz* alleles, two *fz2* alleles, single and double mutant alleles for *otk* and *otk2* and two *Wnt5* alleles were tested. Genetic interactions between *Ror* and *Wnt5* were also analyzed using deficiencies covering both loci.

Genotype	Viability/Phenotype
Ror^{4}/*Ror*^{4}	viable
fz^{J22}/ *fz*^{J22}	viable with PCP defects
Ror^{4}/CyO; *fz*^{J22}/TM6	viable
Ror^{4}/Ror^{4}; *fz*^{J22}/TM6	viable
Ror^{4}/CyO; *fz*^{J22}/ *fz*^{J22}	viable with PCP defects
Ror^{4}/*Ror*^{4}; *fz*^{J22}/ *fz*^{J22} (zygotic)	viable with PCP defects
Ror^{4}/*Ror*^{4}; *fz*^{J22}/ *fz*^{J22}(maternal)	viable with PCP defects
fz^{R52}/ *fz*^{R52}	lethal
Ror^{4}/CyO; *fz*^{R52}/TM6	viable
Ror^{4}/Ror^{4}; *fz*^{R52}/TM6	viable
Ror^{4}/CyO; *fz*^{R52}/ *fz*^{R52}	lethal
Ror^{4}/*Ror*^{4}; *fz*^{R52}/ *fz*^{R52} (zygotic)	lethal
Ror^{4}/*Ror*^{4}; *fz*^{R52}/ *fz*^{R52}(maternal)	lethal
fz^{P21}/ *fz*^{P21}	lethal
Ror^{4}/CyO; *fz*^{P21}/TM6	viable
Ror^{4}/*Ror*^{4}; *fz*^{P21}/TM6	viable
Ror^{4}/CyO; *fz*^{P21}/ *fz*^{P21}	lethal
Ror^{4}/*Ror*^{4}; *fz*^{P21}/ *fz*^{P21} (zygotic)	lethal
Ror^{4}/*Ror*^{4}; *fz*^{P21}/ *fz*^{P21}(maternal)	lethal
fz^{J22}/*fz*^{P21}	viable with PCP defects
Ror^{4}/CyO; *fz*^{J22}/*fz*^{P21}	viable with PCP defects
Ror^{4}/*Ror*^{4}; *fz*^{J22}/*fz*^{P21}(zygotic)	viable with PCP defects
Ror^{4}/*Ror*^{4}; *fz*^{J22}/*fz*^{P21}(maternal)	viable with PCP defects
fz^{J22}/*fz*^{R52}	viable with PCP defects
Ror^{4}/CyO; *fz*^{J22}/*fz*^{R52}	viable with PCP defects

Biology Open • Supplementary information

Ror^{4}/Ror^{4}; fz^{J22}/fz^{R52}(zygotic)	viable with PCP defects
Ror^{4}/Ror^{4}; fz^{J22}/fz^{R52}(maternal)	viable with PCP defects
fz^{P21}/fz^{R52}	viable with PCP defects
Ror^{4}/CyO; *fz^{P21}/fz^{R52}*	viable with PCP defects
Ror^{4}/Ror^{4}; fz^{P21}/fz^{R52}(zygotic)	viable with PCP defects
Ror^{4}/Ror^{4}; fz^{P21}/fz^{R52}(maternal)	viable with PCP defects
Dfz2^{C2}/ Dfz2^{C2}	lethal
Ror^{4}/CyO; *Dfz2^{C2}*/TM6	viable
Ror^{4}/CyO; *Dfz2^{C2}/ Dfz2^{C2}*	lethal
Ror^{4}/Ror^{4}; Dfz2^{C2}/TM6	viable
Ror^{4}/Ror^{4}; Dfz2^{C2}/ Dfz2^{C2}(zygotic)	lethal
Df(3L)469-2/ Df(3L)469-2	lethal
Ror^{4}/CyO; Df(3L)469-2/ TM6	viable
Ror^{4}/CyO; Df(3L)469-2/ Df(3L)469-2	lethal
Ror^{4}/Ror^{4}; Df(3L)469-2/ TM6	viable
Ror^{4}/Ror^{4}; Df(3L)469-2/ Df(3L)469-2 (zygotic)	lethal
Dfz2^{C2}/ Df(3L)469-2	viable (sterile)
Ror^{4}/CyO; *Dfz2^{C2}*/ Df(3L)469-2	viable (sterile)
Ror^{4}/ Ror^{4}; Dfz2^{C2}/ Df(3L)469-2	viable (sterile)
Wnt5^{400}/Wnt5^{400}	viable
Wnt5^{400}/Wnt5^{400}; Ror^{4}/CyO	viable
Wnt5^{400}/Wnt5^{400}; Ror^{4}/Ror^{4}	**lethal**
Wnt5^{400}/Df(1)N19; Ror^{4}/Df(2L)ED729	viable
Wnt5^{400}/Wnt5^{Gal4}; Ror^{4}/Df(2L)ED729	viable
otk^{A1}/ otk^{A1}	viable
otk2^{C26}/ otk2^{C26}	viable
Df(otk,otk2)D72/ Df(otk,otk2)D72	viable (male sterile)
Ror^{4}, otk^{A1}/Ror^{4}, otk^{A1}	viable
Ror^{4}, otk2^{C26}/ Ror^{4},otk2^{C26}	viable

Biology Open • Supplementary information

Ror^{4}*,Df(otk,otk2)D72/ Ror*^{4}*,Df(otk,otk2)D72*	viable (male sterile)

Biology Open • Supplementary information
